# In search of novel ligands using a structure-based approach: a case study on the adenosine A_2A_ receptor

**DOI:** 10.1007/s10822-016-9963-7

**Published:** 2016-09-15

**Authors:** Eelke B. Lenselink, Thijs Beuming, Corine van Veen, Arnault Massink, Woody Sherman, Herman W. T. van Vlijmen, Adriaan P. IJzerman

**Affiliations:** 1 Division of Medicinal Chemistry, Leiden Academic Centre for Drug Research, Leiden University, Einsteinweg 55, 2333 CC Leiden, The Netherlands; 2 Schrödinger, Inc., 120 West 45th Street, New York, NY 10036 USA

**Keywords:** Adenosine A_2A_ receptor, Virtual screening, Explicit water, Novel ligand, Diverse chemical space

## Abstract

In this work, we present a case study to explore the challenges associated with finding novel molecules for a receptor that has been studied in depth and has a wealth of chemical information available. Specifically, we apply a previously described protocol that incorporates explicit water molecules in the ligand binding site to prospectively screen over 2.5 million drug-like and lead-like compounds from the commercially available eMolecules database in search of novel binders to the adenosine A_2A_ receptor (A_2A_AR). A total of seventy-one compounds were selected for purchase and biochemical assaying based on high ligand efficiency and high novelty (Tanimoto coefficient ≤0.25 to any A_2A_AR tested compound). These molecules were then tested for their affinity to the adenosine A_2A_ receptor in a radioligand binding assay. We identified two hits that fulfilled the criterion of ~50 % radioligand displacement at a concentration of 10 μM. Next we selected an additional eight novel molecules that were predicted to make a bidentate interaction with Asn253^6.55^, a key interacting residue in the binding pocket of the A_2A_AR. None of these eight molecules were found to be active. Based on these results we discuss the advantages of structure-based methods and the challenges associated with finding chemically novel molecules for well-explored targets.

**Electronic supplementary material:**

The online version of this article (doi:10.1007/s10822-016-9963-7) contains supplementary material, which is available to authorized users.

## Introduction

**Fig. 1:**
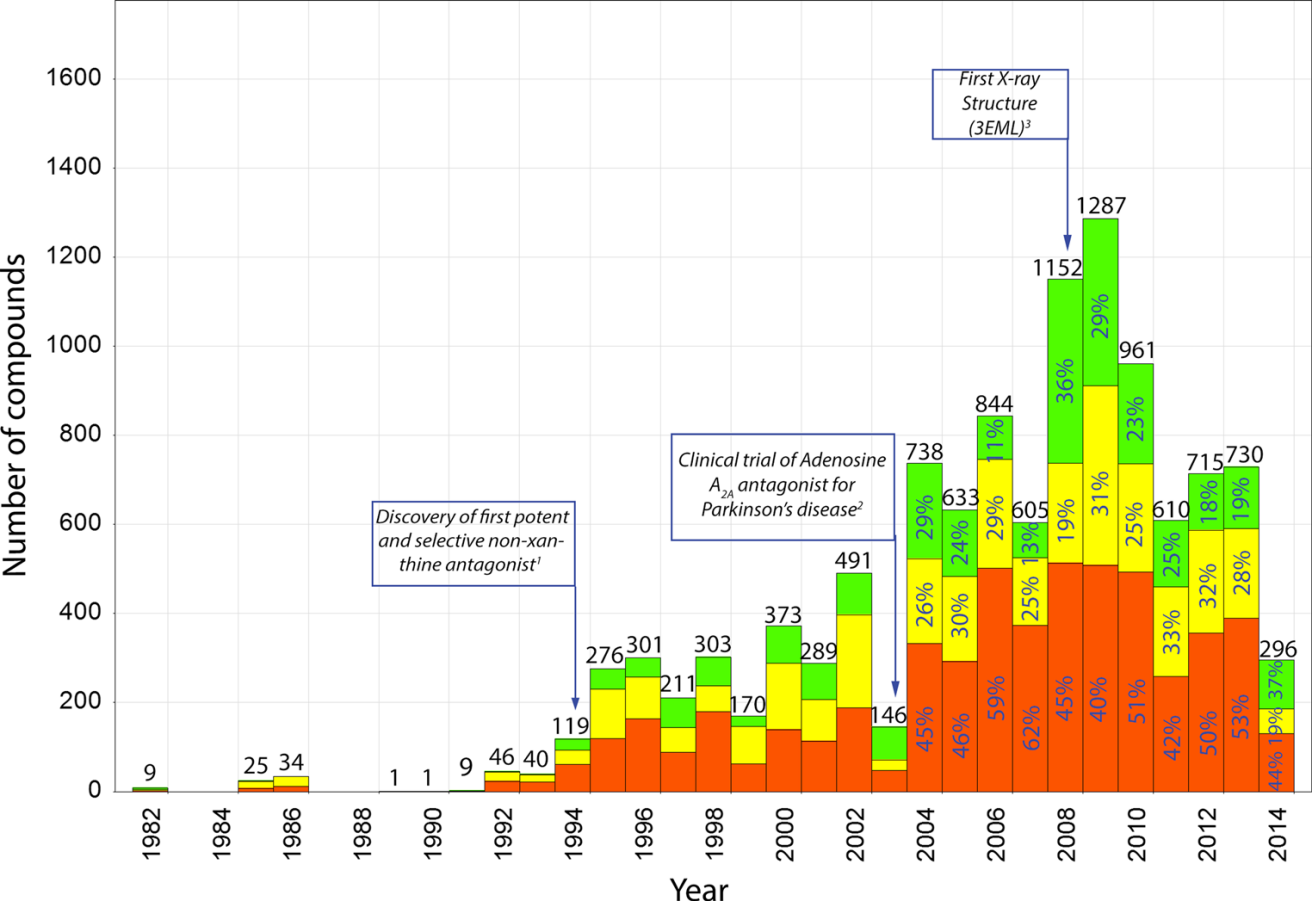
Retrospective overview of bioactivities for the adenosine A_2A_ receptor in ChEMBL (v20). Compounds are colored based on their activity: *green* if the activity (pChEMBL_value) was above 7, *yellow* if it was between 5 and 7, and *orange* if it was below 5 or undefined. The number of tested compounds each year is shown, additionally from 2004 and onwards the percentages for the different categories are shown. The *boxes* shown in the figure indicate key events in adenosine A_2A_ receptor research [1, 4, 44]

Of all members of the class A family of G Protein-Coupled Receptors (GPCRs), the adenosine A_2A_ receptor is one of the best-studied targets. There have been several driving forces behind the interest in this target, such as the discovery of the first potent and selective non-xanthine antagonist (SCH-58261) [1], the discovery of the involvement of the adenosine A_2A_ receptor in Parkinson’s disease [2, 3], and the solving of several early crystal structures. Indeed, one of the first GPCR crystal structures in the Brookhaven Protein Databank was the 2.6 Å crystal structure of the adenosine A_2A_ receptor (PDB: 3EML) [4]. These developments fueled medicinal chemistry research on this receptor, leading to the discovery of many ligands (Fig. 1). Due to the availability of a crystal structure, the adenosine A_2A_ receptor has been a widely studied target using structure-based computational approaches, with at least five independent prospective virtual screening (VS) efforts reported up till now [5–9]. These structure-enabled studies have resulted in the discovery of novel ligand chemotypes such as chromones [5] and 1,2,4-triazines [10]. Most of these VS were based on the first published crystal structure in 2008 of the adenosine A_2A_ receptor [4]. Since the release of this crystal structure, an additional 13 different adenosine A_2A_ receptor structures have been solved, co-crystalized with both agonists and antagonists [11]. The highest resolution crystal structure, with a resolution of 1.8 Å, was released in 2012 (PDB: 4EIY) [12]. Although it was co-crystalized with the same antagonist (ZM-241385) as the first reported structure, it revealed several interesting and novel features, including a large number of water molecules deep in the binding site. These water molecules have been shown to play a pivotal role in binding of ligands to the adenosine A_2A_ receptor [13]. Indeed, redocking of the co-crystalized ligand ZM-241385 was shown to be challenging when no water molecules or restraints were included [14]. The availability of this better solvated high-resolution structure of the adenosine A_2A_ receptor formed the basis to systematically study the influence on virtual screening enrichment of including different water molecules during the docking calculations [15]. In short, we found that five particular water molecules contributed strongly to ligand enrichment, and combining them in an ensemble of receptor structures using a decision tree yielded a screening protocol that showed high enrichments for a retrospective validation dataset.

**Fig. 2:**
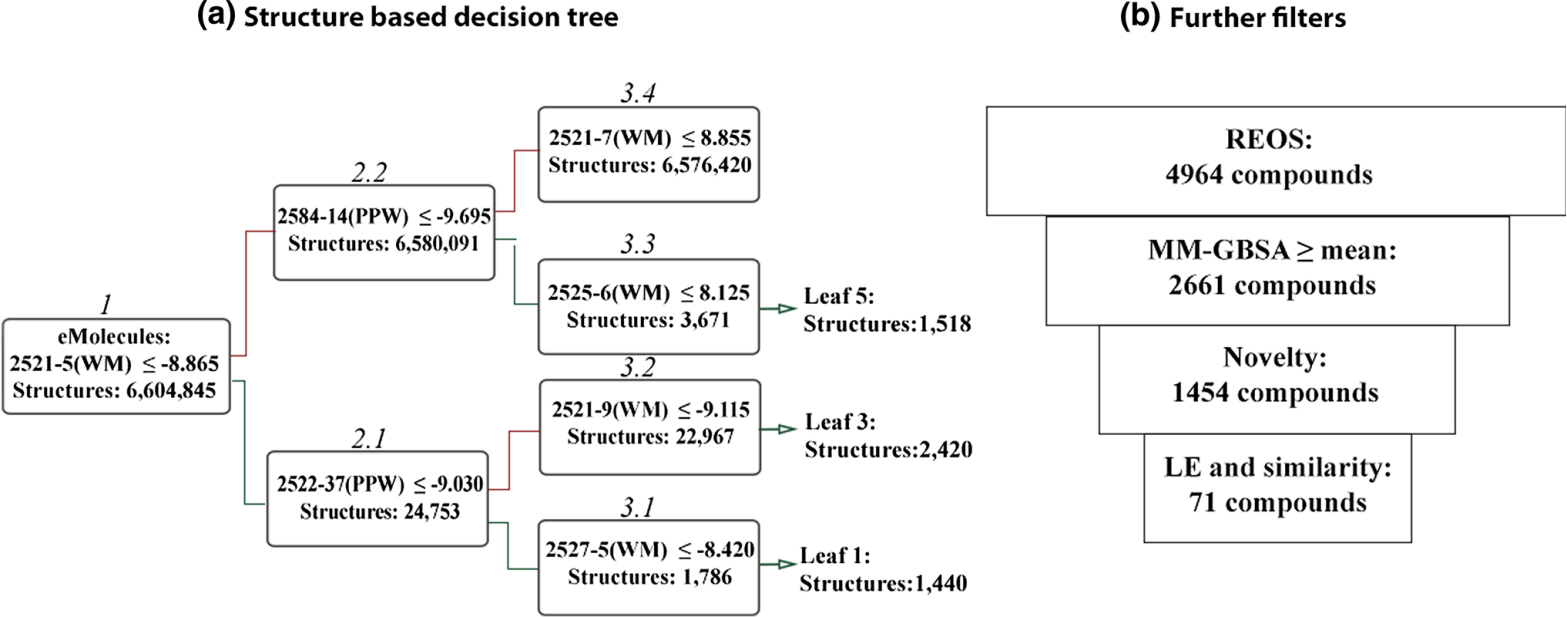
**a** Decision Tree adapted from Lenselink et al. [15] and used here in the virtual screen. Each box represents one docking grid containing the adenosine A_2A_ receptor (PDB: 4EIY) and one explicit binding site water molecule. First, all 6.6M molecules generated by LigPrep and taken from the eMolecules database were docked into the first grid (2521-5 WM). Subsequently, if the docking score cutoff was satisfied molecules proceeded to 2.1 or else to 2.2. Finally, due to their high ratio of ligands versus decoys in the retrospective study, only compounds from leaves 1, 3, and 5 were selected for further inspection. **b** Further filters used in this study to obtain non-reactive and novel compounds

Given the high database enrichment of this protocol validated in our previous retrospective study, here we apply it in a prospective application with the goal of finding novel hits for the A_2A_ receptor. We collected 2.5 M drug-like and lead-like structures from the eMolecules database, and screened them using the VS-decision tree developed previously (Fig. 2). The number of known ligands for the A_2A_ receptor is large (e.g. more than 8800 unique tested compounds reported in ChEMBL) with more than 4000 having an affinity better than 10 µM. Here, we are not interested in discovering additional compounds with typical A_2A_AR binding features, but rather we aim to discover truly novel scaffolds for this receptor. Thus, after filtering the top computational hits in our protocol and excluding compounds with similarity to previously tested compounds, we selected and acquired a total of 71 compounds. Later we added 8 additional compounds that were selected for making a key interaction with the receptor, after further visual inspection of the docking results. These compounds were then evaluated for their affinity to the adenosine A_2A_ receptor in a radioligand binding displacement assay. In total two novel compounds were found. In this work, the challenging nature of finding chemically novel molecules for well-explored targets is discussed.

## Methods

### Virtual Screen

All structural modeling was performed using tools in the Schrödinger small-molecule discovery suite. Docking was done with Glide 6.3 [16] using the SP scoring function with default settings. We used a previously generated ensemble of A_2A_ receptor models prepared with the Protein Preparation Wizard [17], each containing different individual water molecules [15]. The eMolecules database was prepared using LigPrep with default settings. Protonation and tautomeric states were assigned using Epik [18, 19]. This resulted in a fully expanded set of ~6.6 M stereoisomeric and tautomeric states from the 2.5 M ligands. The fully expanded set of molecules was docked into the upper node of the decision tree (DT) (i.e. the one that generated the highest enrichment in our previous study). Ligands proceeded through the decision tree according to previously defined rules (Fig. 2) based on Glide docking scores. For instance in order to end up in leaf 3, compounds should have a docking score better than -8.865 kcal/mol in the first node, a docking score better than −9.030 kcal/mol in the second and better than −9.115 in the third node of the decision tree. The compounds that ended up in leaf 1, leaf 3, and leaf 5 (5378 total) were considered to be “computational hits” and were subjected to subsequent filters (Fig. 2).

First we eliminated reactive compounds using the REOS filter (as implemented in KNIME) [20, 21]. Next, we rescored all poses using the MM-GBSA method [22], where we used the VSGB 2.0 implicit solvent model [23] and the OPLS2005 force field [24] to estimate a binding energy. For the MM-GBSA calculations, explicit water molecules were not considered. All compounds with a MM-GBSA binding energy worse than the mean were eliminated. Since our goal was to find novel scaffolds, we included an explicit similarity filter. This filter was based on *all* compounds ever tested on the A_2A_AR for activity from ChEMBL v17 (human, rat, mouse, bovine, guinea pig, sheep, rabbit, and pig), resulting in a total of 12,205 compounds. Tanimoto similarities between all computational hits and all tested compounds were calculated based on Molprint2D [25] fingerprints in Canvas [26] and computational hits were eliminated if the similarity to *any* of the compounds in ChEMBL was higher than a defined threshold (Tanimoto >0.25) [27]. Next, we constructed two different sets: A) 71 compounds from the previous steps, ranked by solvent accessible ligand efficiency (LE^2/3^) [28] and filtered iteratively (based on the rank) by similarity within the selection of compounds (Tanimoto ≤0.25), and B) 8 compounds from the previous steps, prior to the filtering and ranking within the selection of computational hits (before step A), based on both visual inspection and further filtering to ensure a bidentate interaction with Asn253^6.55^ in the 6th transmembrane helix of the receptor was present.

### Cheminformatics

**Fig. 3:**
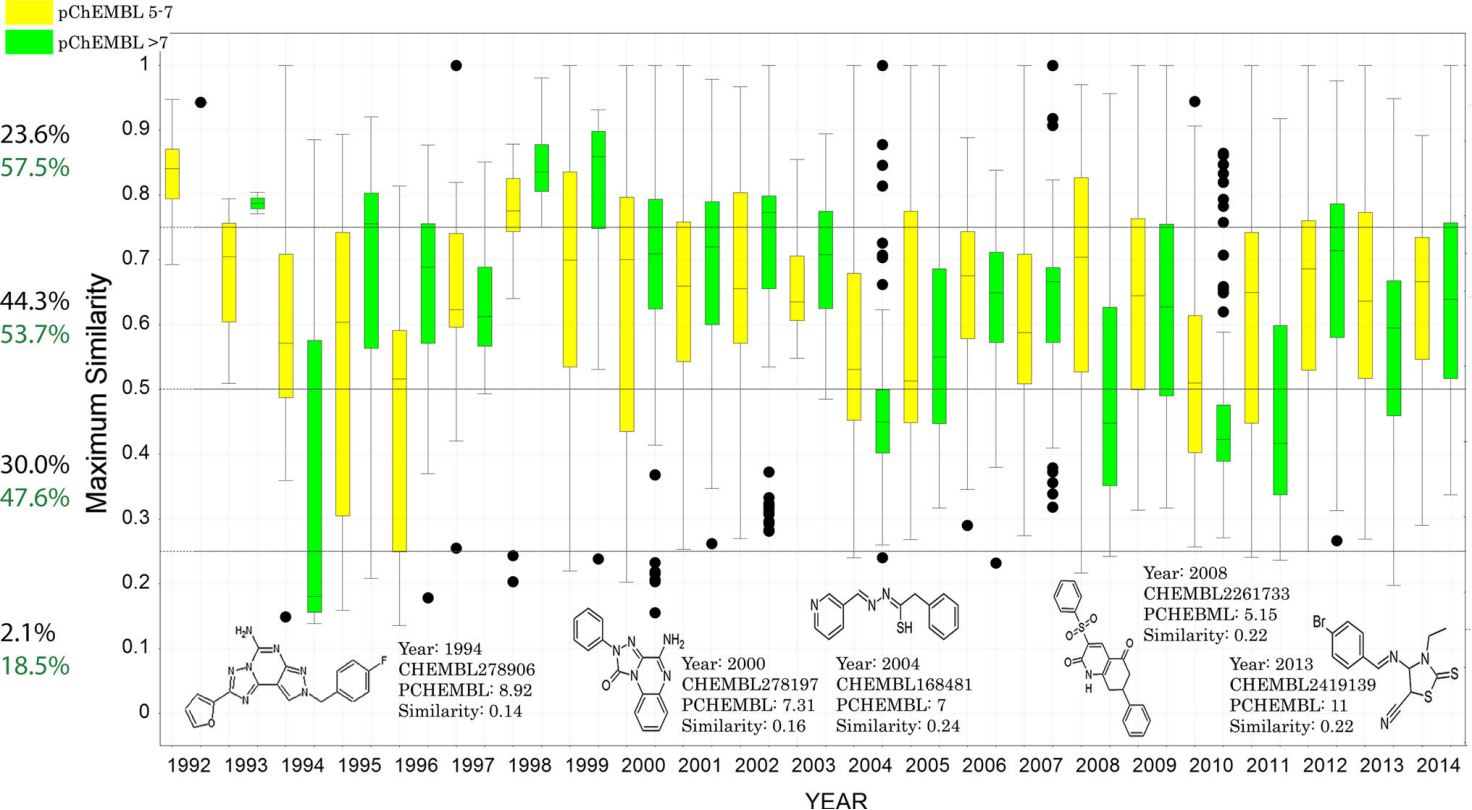
Retrospective overview of ligand discovery for the adenosine A_2A_ receptor. Similarity represents the maximum similarity against all previously reported compounds (see “Methods” section). *Bars* are colored based on activity: *yellow bars* represent compounds that have relatively low affinity (pChEMBL 5–7), compounds in green are more potent (pChEMBL > 7). Inactive compounds (pChEMBL < 5) are not shown. The *black percentages* represent the distribution of compounds in the four different novelty bins. *Green percentages* represent the percentage of active compounds (pChEMBL > 5) in that bin. Several examples of diverse compounds (similarity ≤ 0.25) are shown

**Fig. 4:**
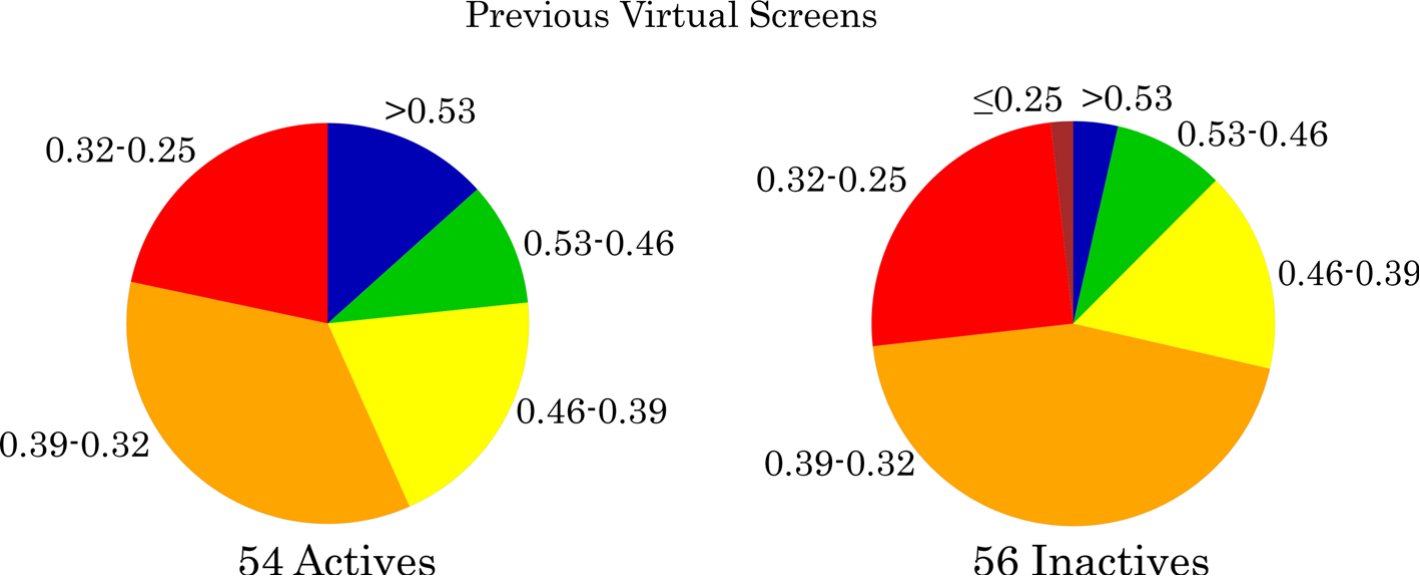
Similarity of compounds from five previous reported virtual screens [6–10]. Both for inactive (*right*) and active compounds (*left*) a pie chart is shown, in which compounds are distributed into different similarity bins based on a step size of 0.07. For the inactives, compounds with a low similarity (≤0.25, *dark red*) and high similarity (<0.53, *blue*) are represented by one bin each. For the actives no compounds were found in the low similarity bin (≤0.25)

Molprint2D [25] fingerprints, as implemented in Canvas [26, 29], were used for similarity calculations. While the filtering screen was performed using ChEMBL v17, we retrospectively updated the dataset from ChEMBL v17 to v20 to include more data in the analysis figures (Figs. 1, 3). All duplicate molecules were removed. In order to generate Fig. 3, for each compound for the A_2A_AR in ChEMBL, a global Tanimoto-based similarity score was calculated by comparing the maximum similarity for a given compound to any of compounds (including inactive compounds) reported previously to the compound in question. This yielded a similarity score for every compound, and in this way we were able to visualize the global similarity of 4501 ligands using a bar chart. Figures were generated using Dotmatics Vortex v2015.06.41692. For the analysis of previously performed VS (Fig. 4), compounds were extracted from ChEMBL if available, or else manually retrieved from the reporting articles.

### Radioligand binding assays

[^3^H]ZM241385 (45.9 Ci/mmol) was purchased from ARC Scopus Research (Wageningen, The Netherlands). NECA was obtained from Sigma-Aldrich (St. Louis, MO, USA). The compounds selected by the VS were bought from Enamine, Vitas M, Chembridge, Chemdiv and Key Organics. All other materials were purchased from commercial sources.

HEK293 cells stably expressing human adenosine A_2A_ receptor were cultured and harvested as has been described previously [30]. Single point radioligand displacement assays were performed in order to determine radioligand displacement by the purchased molecules. 2.5 nM [3H]ZM241385 was incubated for 2 h. at 25 °C in a final volume of 100 μL with 15 µg A_2A_AR-WT membranes, in absence or presence of 10 μM of the purchased molecules. Total binding (TB) was determined in the absence of the purchased compounds and non-specific binding (NSB) was measured in the presence of 100 µM NECA. The amount of protein added ensured that TB was less than 10 % of the total radioactivity added to prevent radioligand depletion. Incubations were terminated by rapid vacuum filtration to separate the bound from free radioligand through 96-well GF/B filter plates using a FilterMate™ Harvester (PerkinElmer Life Sciences). Filters were subsequently washed ten times with ice-cold assay buffer and left to dry for at least 15 min at 55 °C. Thereafter, 25 µL of scintillation fluid (PE Microscint 20) was added and after at least 3 h the filter-bound radioactivity was measured by scintillation spectrometry using a PE 2450 MicroBeta2 Plate Counter (PerkinElmer Life Sciences). In order to determine the total counts and thus the exact concentration of radioligand, 3.5 mL of PE Emulsifier Safe was added to 25 µL of radioligand and the radioactivity was measured with the PE Liquid Scintillation Analyzer Tri-Carb 2900TR (PerkinElmer Life Sciences). The experiments were performed two times in triplicate. The experimental data was analyzed with GraphPad Prism 5.0 (GraphPad Software Inc., San Diego, CA, USA).

## Results

### Experimental Results

After the initial virtual screen (VS) was performed, we selected compounds from leaves 1, 3, and 5 because they were previously shown to contain the majority of ligands in our retrospective validation (Fig. 2) [15]. These compounds, totaling 5378, were further filtered to ensure a selection of diverse and non-reactive compounds (Fig. 2). All selected compounds (1454) were novel, where novelty was defined as having a Tanimoto similarity below or equal to 0.25 to all active A_2A_AR ligands (including non-actives). 71 compounds were purchased and tested in a radioligand binding displacement assay for adenosine A_2A_ receptor affinity (see Methods). Two hits were found (Table 1) that met the criterion of approximately 50 % radioligand displacement at a concentration of 10 μM. These two hits have little similarity with the known active ligands in ChEMBL (see Table 1). The first compound only shares the furan and amide moieties with the most similar known A_2A_AR active compound, thus representing a newly identified scaffold. The second scaffold has only an amide in common with one of the active A_2A_AR compounds from ChEMBL.

**Table 1:** Chemical structures of the two hits identified in this study

2D Structure	Displacement at 10 μM (%)^a^	ChEMBL^b^	T_c_^b^	Docking score (kcal/mol) Rank^c^
	73 ± 1		0.23	−9.17 5326
	47 ± 1		0.23	−9.59 1530

^a^Percentage displacement of ZM-241385 at the adenosine A_2A_ receptor at 10 μM of the tested ligand

^b^Closest neighbor in ChEMBL with adenosine A_2A_ receptor activity, and Tanimoto similarity (T_c_) of the closest neighbor

^c^The rank and docking score of both ligands in the first node of the decision tree

Aside from these two compounds, none of the other molecules showed more than 50 % radioligand displacement (SI Table 1). To make sure our results were not caused by the over rewarding of non-native interactions by Glide, we reevaluated the binding poses of the selected 1454 compounds, and compared these with the binding mode of adenosine A_2A_ receptor ligands for which the binding mode was known. Indeed the poses of both the two actives and top scoring inactives are arguably overlapping, with all 7 compounds interacting with Asn253^6.55^ (SI Figures 1 and 2, poses of the 2 actives and 5 top scoring non-actives). However, we observed that known active compounds tended to form a bidentate H-bond with amino acid Asn253^6.55^, a residue crucial for ligand binding, whereas most of our top scoring virtual screening hits did not. Based on the 1454 compounds, a second selection of 8 compounds was made to ensure that these compounds form this critical interaction as well. These compounds were also tested in the same displacement assay; however none of them showed radioligand displacement above 50 % (SI Table 2).

### Analysis of other compounds found in the Virtual Screen

To determine the ability of the docking algorithm to identify active compounds irrespective of novelty, we analyzed the compounds that were discarded based on the similarity criterion. In total this group represented 1207 compounds. We grouped these compounds based on the corresponding most similar compound found in ChEMBL tested for activity on the adenosine A_2A_ receptor. Frequencies of compounds most similar to corresponding ChEMBL compounds were generated in order to determine how the compounds would cluster, e.g. what type of compounds would be frequently occurring in our VS (Table 2). For instance, in the first row is the most frequently occurring compound, ChEMBL2030685, which has a pChEMBL value of 5.53 and was previously identified by Langmead et al. [31]. The pChEMBL represents the negative log of concentration–response activity values (e.g. IC50/EC50/Ki/Kd). In our screen we found 71 compounds that were most similar to ChEMBL2030685, with the highest Tanimoto similarity being 0.44. We found that many compounds bear structural resemblance to compounds found in ChEMBL, many of which were previously identified using in silico methods (highlighted in bold). For instance, two compounds were identical to ligands previously identified by Sanders et al. (ChEMBL2070726 and ChEMBL1714515) [32]. In total the top ten most frequent occurring compounds of ChEMBL represented 402 of the 1207 compounds (~33 %).

**Table 2:** Ten most frequent occurring ChEMBL ligands when compared with the compounds identified in our screen

ChEMBL	pChEMBL^a^	Source^a^	Frequency^b^	Most similar^b^	T_c_^b^
	5.53	Langmead et al. [5]	71		0.44
	–	Novellino et al. [45]	68		0.47
	5.54	Katritch et al. [6]	48		0.48
	–	Katritch et al. [6]	45		0.58
	5.6	Sanders et al. [32]	37		1.00
	5.3	Sanders et al. [32]	33		1.00
	6.52	Carlsson et al. [33]	27		0.62
	6.19	Poulsen et al. [46]	27		0.41
	–	van Muijlwijk-Koezen et al. [47]	26		0.41
	5.7	Langmead et al. [5]	20		0.28

^a^Affinity values are given if the compound was active against the adenosine A_2A_ receptor (pChEMBL_value). The source lists the publication where the compounds originate from, in bold are publications where the source of compounds is commercial and the compound was found using a computational method

^b^Frequencies represent the number of times a certain ChEMBL-ligand is identified as the most similar in the discarded novelty bins (<0.75), e.g. ChEMBL2030685 (row 1) is most similar to 71 compounds in our screen. The most similar compound in our screen is shown and Tanimoto similarity (T_c_) indicates the similarity value of this compound to the reference ChEMBL compound

Also apparent in Table 2 is that certain substructures appear frequently; for example the succinimide moiety is found in 3 of the 10 entries shown. When we analyzed how frequently this substructure occurs in the raw results (the 5378 compounds prior to filtering) we found that it appeared 411 times (7.6 %), possibly because it is often used as a protective group in synthesis. Although in the docking the oxygen of the succinimide was often found to make an interaction with the backbone of Phe168^EL2^ (e.g. SI Figure 2), no evidence was found that it was a beneficial group. First of all, more than 60 % of the computational hits (prior to filtering) containing a succinimide were found to have an MM-GBSA score worse than mean (254 of the 411 of compounds). Moreover, when we queried compounds tested on the adenosine A_2A_ receptor (ChEMBL v20) for this substructure mostly inactive compounds were found (37 compounds with pChEMBL < 5, compared to 0 highly active compounds (pChEMBL > 7), and 16 compounds with a moderate activity (pChEMBL value between 5 and 7)). To put this in context, the 1,3,5-triazine moiety found in reference antagonist ZM-241385 is only present in 11 of our potential hit compounds (0.2 %), versus 166 highly active ligands in ChEMBL. When we further analyzed the source of these compounds we found that, of the top 10 most frequently occurring ChEMBL compounds, 7 originated from computational studies. Additionally, although ChEMBL2151129 (Table 2, row 7) described by Carlsson et al. [33] was synthesized, the original scaffold was discovered using a structure-based VS [7]. These results indicate that of the not-so-novel compounds discarded in our screen, many represent compounds found by computational methods that have been experimentally confirmed as A_2A_AR actives.

### Retrospective analysis of the adenosine A_2A_ receptor Ligand Discovery

The challenging nature of finding non-similar molecules in a virtual screen, for a thoroughly studied target, resulted in the identification of two hit compounds in this study. Therefore we compared our similarity threshold with the similarity of compounds tested on the adenosine A_2A_ receptor. All compounds tested for A_2A_ receptor binding in ChEMBL were sorted by year of publication, and a global maximum similarity to earlier reported compounds was calculated to categorize whether bioactive compounds were novel at the time of reporting.

Overall, the distribution of compounds across the different novelty bins seems to be skewed toward similarity over novelty, with a mean similarity of 0.6 and around 68 % (44.3 + 23.6 %, percentages shown in black, Fig. 3) of the compounds falling in the two bins above 0.5 similarity. The lowest similarity bin was the least populated, in which only 93 out of the 4501 active ligands were present (2.1 %). This is also demonstrated by the fact that in the majority of years, the bar charts do not cross the ≤0.25 similarity line. Some of the novel scaffolds that have been reported include the pyrazolo [4,3-e] 1,2,4-triazolo [1,5-c]pyrimidines in 1994, 1,2,4-triazolo [4,3-a]quinoxalin-1-ones in 2000, and 2-thioxothiazole derivatives in 2013 [1, 34, 35] (Fig. 3).

The 93 active ligands in the lowest similarity bin constitute, using the current definition, the total number of unique scaffolds that have been discovered over the years. Next we compared the number of actives as a fraction of the total number of reported compounds, across the different similarity bins (percentages shown in green). This confirms the expected result that the less similar a newly tested compound is, the lower the chance is that it will be active. However for active compounds, we did not find a significant difference between active (green, pChEMBL >7) and less highly active (yellow, pChEMBL 5–7) compounds, although this seems to vary strongly between years. Nevertheless, in the lowest similarity bin less than 1 in 5 compounds (18.5 %) was found to be active, thus demonstrating the difficulty in finding molecules that are both active (18.5 %) and novel (2.1 %) for a well-studied target with many actives.

### Retrospective analysis of Virtual Screens

Because at least five structure-based VS have been conducted on the adenosine A_2A_ receptor prior to the VS reported here, we assumed that similar trends (as in Fig. 3) could be observed for these earlier screens as well [6–10]. To test this hypothesis we retrieved all the compounds from the five known screens that were reportedly bought and tested (unfortunately, not all the papers reported full datasets of all tested compounds). The different screens yielded a combined total of 54 active compounds. When we compared these active molecules with the reported inactive compounds from those studies we learned that, similar to the retrospective analysis shown in Fig. 3, there is a trend toward lower similarity amongst the inactive compounds. For instance, almost 75 % of the reported inactives had a similarity ≤0.39 while only 55 % of the active compounds were found in these bins. There is a higher fraction of low similarity compounds reported in the five VS than in the retrospective data, e.g. more than 75 % of the actives from these VS had a similarity below 0.50 (Fig. 4) compared with 32 % for the retrospective data (Fig. 3; 30 + 2.1 % respectively). Nevertheless none of the actives found with VS were below the 0.25 similarity threshold, and using our current definition they cannot be considered novel scaffolds. The three most novel compounds originated from the VS by Heptares [5] where the authors explicitly selected non-similar molecules (hit 3, hit 4, and hit 9 in their publication) [5].

## Discussion

In this study we used a previously established structure-based virtual screening protocol [15] that incorporates explicit water molecules to find novel ligands for the adenosine A_2A_ receptor. A more practical scenario would involve pre-filtering based on 2D-similarity and reactivity (REOS), increasing efficiency of the screen; however docking of all compounds allowed us to compare the non-similar/novel computational hits with the full spectrum of docked compounds. We defined ‘novel’ as compounds with a Tanimoto similarity smaller than or equal to 0.25 compared to any known compound tested for activity on the adenosine A_2A_ receptor. Our hit rate was much lower (1.4 %) than in previously reported screens and we determined that the low hit rate could be most likely attributed to an overly stringent requirement to find novel compounds for a target that has a wealth of chemical matter. In retrospect a higher cutoff around the mean similarity (0.40) of adenosine A_2A_AR compounds (see “Retrospective analysis of the adenosine A_2A_ receptor Ligand Discovery”) might have been more suitable. Because of the use of different fingerprints, similarity thresholds in other VS are only comparable qualitatively. Nonetheless, we can draw some conclusions about the results obtained here when compared with those from previous screens. In the study by Katritch et al. [6], who also included explicit water molecules in their VS, a hit rate of 41 % (23/56) at a test concentration of 10 µM was observed. Hits were compared with molecules in GLIDA [36], and their calculated similarities ranged from 0.44 to 0.68 [6]. Another study by Carlsson et al. where modified partial charges were used on Asn253^6.55^ to favor hydrogen bonding to this residue yielded a comparable hit rate of 35 % (7/20) at a test concentration of 20 µM [7]. Moreover, the authors employed filters such as molecular weight, formal charges, and the Similarity Ensemble Approach (SEA) to further narrow down their hit list. Compounds emerging from this screening approach were compared with adenosine receptor ligands from ChEMBL and Wombat, revealing similarities between 0.30 and 0.52. A subsequent study by Langmead et al. [5] was performed on a homology model of the adenosine A_2A_ receptor yielding a hit rate of 9 % (20/230) at a test concentration of 55 µM. Compounds were filtered for CNS-like properties and ligands containing xanthine or furan moieties were removed. Subsequent hits were subjected to a biophysical mapping approach [13]. The 10 hits that were reported all had relatively low similarities to known A_2A_AR antagonists, ranging between 0.19 and 0.31. However the authors noted that their most potent scaffold contained the same moiety as found in the previous two virtual screens described above [6, 7]. Another recent screen focused on finding fragments for the adenosine A_2A_ receptor [8] resulted in a hit rate of 64 % (14/22) at a test concentration of 500 µM, while similarities to known compounds ranged between 0.28 and 0.41. Lastly, a VS was performed by Rodriguez et al. to identify agonists for the adenosine A_2A_ receptor [9]. Although none of the hits turned out to be agonists, a hit rate of 45 % (9/20) was achieved at a test concentration of 10 µM, with similarities ranging from 0.30 to 0.61.

In this study we attempted to determine the ability of docking-based VS to identify novel compounds. We explicitly selected compounds that bore no resemblance to any compound previously tested against the adenosine A_2A_ receptor. Indeed, the adenosine A_2A_ receptor is one of the best studied targets of all class A GPCRs, and the number of bioactivities (10,184) in ChEMBL (version 20) ranks it at number 7 of the 688 class A GPCRs [37]. This represents the result of decades of screening and medicinal chemistry efforts (see also Figs. 1, 3) [38]. In our screen we included not only active but also inactive compounds as similarity filters. This was done primarily to exclude inactive molecules identified in previous screens, but also to explore actual uncharted chemical space. In some VS [5, 8] not all the tested compounds, i.e. including inactives, have been disclosed. This arguably leads to a loss of useful information, in the form of validated ‘true negatives’ that can be used as decoys in a benchmark screen or to bias future screening efforts away from explored chemical space. As a consequence of this data incompleteness we may have tested compounds that had been found inactive in previous studies as well. Including this information in future VS reports would be helpful to prevent future screens from exploring the same (inactive) chemical space.

Despite the differences in data completeness, we attempted to compare previous VS results with our current similarity-filtered method. We found that ligands identified in a VS have a higher novelty than the ligands for the adenosine A_2A_ receptor (Figs. 3, 4) in general. Inactives have an even higher novelty, which is the consequence of a tradeoff between similarity and activity. In fact it is a well-established paradigm that activity correlates with similarity [23, 24, 39]. Indeed, this is the basis for 2D virtual screening. Therefore depending on the goal of VS, and the extent of the mapped chemical space, one may wish to filter out molecules with a novelty *above* a certain threshold (e.g. ≥0.75) as the chances of success become increasingly small as similarity to known actives decreases. On this basis, virtual screening efforts often include a step of visual inspection, where ligands that interact in non-native manner (e.g. no hydrogen bond with key residue) are filtered out [40]. Selecting compounds across a range of similarity, e.g. between 0.20 and 0.50 provides a good balance between potential activity and novelty (Fig. 3). Another possibility would be to test compounds that have a higher novelty at a higher concentration. In this study we considered hits as displaying approx. 50 % or higher radioligand displacement at 10 µM (equivalent to pChEMBL >5), but in previously performed VS concentrations between 10 and 500 µM were used as a test concentration. Additionally the number of selected compounds based on all the filters (Fig. 2) is on the low side due to the relatively high docking score cutoffs in the decision tree. These scores were determined based on known and potent ligands [15], and in hindsight these cutoffs could have been adjusted to select hits from a larger pool of compounds. Indeed, it is crucial to select ligands that end up in multiple leaves to capture more chemical space [15]. Here we selected compounds that satisfied the docking score cutoff in at least two out of the three nodes (i.e. compounds ending up in leaf 1, 3, and 5, Fig. 2a). This could easily be extended to another ensemble strategy such as the “Z2 score”, which combines Z-scores from multiple screens and has been proven to outperform other ensemble methods [41].

Although some of the virtual screens (by us and others) have expanded A_2A_ receptor chemical space with new chemotypes, they all draw from essentially the same pool of commercially available compounds (Table 2). In addition it has been shown that available chemical libraries only cover a limited part of the chemical space [42, 43]. Future studies using unbiased libraries that cover larger chemical spaces such as GDB [42] could potentially suggest new chemotypes for this important target.

## Conclusions

Structure-based virtual screening is a widely used approach to identify hits for drug targets, and has been applied previously to the adenosine A_2A_ receptor. We performed a virtual screen on this target while excluding all compounds similar to known actives and inactives in order to explore unchartered chemical space for this GPCR. Of the 79 compounds we tested, only 2 exhibited affinity for the target. We found several explanations for this relatively low hit rate: the overly stringent novelty threshold, the abundance of known ligands for the adenosine A_2A_ receptor, and the overlap in results with previous virtual screening efforts. Finding novel compounds for a particular target of interest becomes increasingly difficult as the target is more extensively studied, as was the case with the adenosine A_2A_ receptor studied here. Nonetheless, structure-based methods still seem to offer the greatest possibility to find novel actives for targets that have been well explored, since ligand-based methods are generally designed to find compounds similar to known actives. As such, researchers should choose their virtual screening method for a project based on their objectives and the known chemical matter for the target. Structure-based methods may not be necessary when sufficiently interesting compounds can be found with simpler/faster methods. However, when minimal biases from existing chemical matter is sought, structure-based methods will likely be preferred, even though there is still substantial room for these methods to improve based on treating the underlying physics of the system more accurately (protein flexibility, explicit waters, variable ionization states, etc.). With improvements of structure-based virtual screening methods coupled with rapidly increased computational capabilities to handle the complexities of sampling we expect to see the value of these methods continue to increase in the coming years.

## Supporting information

Displacement results of the non-active molecules, docking poses of the two actives and five top scoring inactives, HPLC and ^1^H NMR spectra of the active molecules.

## Electronic supplementary material

Below is the link to the electronic supplementary material.

Supplementary material 1 (DOCX 13675 kb)

## References

[1] Baraldi PG, Manfredini S, Simoni D, Zappaterra L, Zocchi C, Dionisotti S, Ongini E (1994). Bioorg Med Chem Lett.

[2] Fuxe K, Ferré S, Snaprud P, von Euler G, Johansson B, Ferdholm B (1993). Drug Dev Res.

[3] Jenner P (2003). Neurology.

[4] Jaakola VP, Griffith MT, Hanson MA, Cherezov V, Chien EY, Lane JR, IJzerman AP, Stevens RC (2008). Science.

[5] Langmead CJ, Andrews SP, Congreve M, Errey JC, Hurrell E, Marshall FH, Mason JS, Richardson CM, Robertson N, Zhukov A (2012). J Med Chem.

[6] Katritch V, Jaakola VP, Lane JR, Lin J, IJzerman AP, Yeager M, Kufareva I, Stevens RC, Abagyan R (2010). J Med Chem.

[7] Carlsson J, Yoo L, Gao ZG, Irwin JJ, Shoichet BK, Jacobson KA (2010). J Med Chem.

[8] Chen D, Ranganathan A, IJzerman AP, Siegal G, Carlsson J (2013). J Chem Inf Model.

[9] Rodríguez D, Gao Z-G, Moss SM, Jacobson KA, Carlsson J (2015). J Chem Inf Model.

[10] Congreve M, Andrews SP, Dore AS, Hollenstein K, Hurrell E, Langmead CJ, Mason JS, Ng IW, Tehan B, Zhukov A, Weir M, Marshall FH (2012). J Med Chem.

[11] Katritch V, Cherezov V, Stevens RC (2013). Annu Rev Pharmacol Toxicol.

[12] Liu W, Chun E, Thompson AA, Chubukov P, Xu F, Katritch V, Han GW, Roth CB, Heitman LH, IJzerman AP, Cherezov V, Stevens RC (2012). Science.

[13] Bortolato A, Tehan BG, Bodnarchuk MS, Essex JW, Mason JS (2013). J Chem Inf Model.

[14] Ivanov AA, Barak D, Jacobson KA (2009). J Med Chem.

[15] Lenselink EB, Beuming T, Sherman W, van Vlijmen HW, IJzerman A (2014). J Chem Inf Model.

[16] Friesner RA, Banks JL, Murphy RB, Halgren TA, Klicic JJ, Mainz DT, Repasky MP, Knoll EH, Shelley M, Perry JK, Shaw DE, Francis P, Shenkin PS (2004). J Med Chem.

[17] Sastry GM, Adzhigirey M, Day T, Annabhimoju R, Sherman W (2013). J Comput Aided Mol Des.

[18] Shelley JC, Cholleti A, Frye LL, Greenwood JR, Timlin MR, Uchimaya M (2007). J Comput Aided Mol Des.

[19] Greenwood JR, Calkins D, Sullivan AP, Shelley JC (2010). J Comput Aided Mol Des.

[20] Berthold MR, Cebron N, Dill F, Gabriel TR, Kötter T, Meinl T, Ohl P, Sieb C, Thiel K, Wiswedel B (2008). KNIME: the Konstanz information miner.

[21] Walters WP, Stahl MT, Murcko MA (1998). Drug Discov Today.

[22] Schrödinger Release 2013-3: Prime v, Schrödinger, LLC, New York, NY (2013)

[23] Li J, Abel R, Zhu K, Cao Y, Zhao S, Friesner RA (2011). Proteins Struct Funct Bioinf.

[24] Banks JL, Beard HS, Cao Y, Cho AE, Damm W, Farid R, Felts AK, Halgren TA, Mainz DT, Maple JR, Murphy R, Philipp DM, Repasky MP, Zhang LY, Berne BJ, Friesner RA, Gallicchio E, Levy RM (2005). J Comput Chem.

[25] Bender A, Mussa HY, Glen RC, Reiling S (2004) Similarity searching of chemical databases using atom environment descriptors (MOLPRINT 2D): evaluation of performance. J Chem Inf Comput Sci 44:1708–1718 10.1021/ci0498719 15446830

[26] Sastry M, Lowrie JF, Dixon SL, Sherman W (2010). J Chem Inf Model.

[27] Babine RE, Bender SL (1997). Chem Rev.

[28] Loving K, Alberts I, Sherman W (2010). Curr Top Med Chem.

[29] Duan J, Dixon SL, Lowrie JF, Sherman W (2010). J Mol Graphics Modell.

[30] Massink A, Gutiérrez-de-Terán H, Lenselink EB, Zacarías NVO, Xia L, Heitman LH, Katritch V, Stevens RC, IJzerman AP (2015). Mol Pharmacol.

[31] Zhukov A, Andrews SP, Errey JC, Robertson N, Tehan B, Mason JS, Marshall FH, Weir M, Congreve M (2011). J Med Chem.

[32] Sanders MP, Roumen L, van der Horst E, Lane JR, Vischer HF, van Offenbeek J, de Vries H, Verhoeven S, Chow KY, Verkaar F, Beukers MW, McGuire R, Leurs R, IJzerman AP, de Vlieg J, de Esch IJ, Zaman GJ, Klomp JP, Bender A, de Graaf C (2012). J Med Chem.

[33] Carlsson J, Tosh DK, Phan K, Gao Z-G, Jacobson KA (2012). ACS Med Chem Lett.

[34] Colotta V, Catarzi D, Varano F, Cecchi L, Filacchioni G, Martini C, Trincavelli L, Lucacchini A (2000). J Med Chem.

[35] Mishra CB, Sharma D, Prakash A, Kumari N, Kumar N, Luthra PM (2013). Bioorg Med Chem.

[36] Okuno Y, Tamon A, Yabuuchi H, Niijima S, Minowa Y, Tonomura K, Kunimoto R, Feng C (2008). Nucleic Acids Res.

[37] Gaulton A, Bellis LJ, Bento AP, Chambers J, Davies M, Hersey A, Light Y, McGlinchey S, Michalovich D, Al-Lazikani B, Overington JP (2012). Nucleic Acids Res.

[38] Fredholm BB, IJzerman AP, Jacobson KA, Linden J, Müller CE (2011). Pharmacol Rev.

[39] Muchmore SW, Debe DA, Metz JT, Brown SP, Martin YC, Hajduk PJ (2008). J Chem Inf Model.

[40] Irwin JJ, Shoichet BK (2016) J Med Chem 12;59(9):4103 10.1021/acs.jmedchem.5b02008 PMC4865415 26913380

[41] Sastry GM, Inakollu VS, Sherman W (2013). J Chem Inf Model.

[42] Ruddigkeit L, Van Deursen R, Blum LC, Reymond J-L (2012). J Chem Inf Model.

[43] Reymond J-L (2015). Acc Chem Res.

[44] Bara-Jimenez W, Sherzai A, Dimitrova T, Favit A, Bibbiani F, Gillespie M, Morris M, Mouradian M, Chase T (2003). Neurology.

[45] Novellino E, Cosimelli B, Ehlardo M, Greco G, Iadanza M, Lavecchia A, Rimoli MG, Sala A, Da Settimo A, Primofiore G (2005). J Med Chem.

[46] Poulsen S-A, Young DJ, Quinn RJ (2001). Bioorg Med Chem Lett.

[47] van Muijlwijk-Koezen JE, Timmerman H, Vollinga RC, Frijtag von Drabbe Künzel J, de Groote M, Visser S, IJzerman AP (2001). J Med Chem.

