# Supplementary material for: In search of novel ligands using a structure-based approach: a case study on the adenosine A2A receptor
Source: J Comput Aided Mol Des. 2016 Sep 15;30(10):863–74. doi: 10.1007/s10822-016-9963-7 (PMC5083784; doi:10.1007/s10822-016-9963-7)

*Supplementary Information*

In search of novel ligands using a structure-based approach – A case study on the adenosine A_2A_ receptor.

Authors: Eelke B. Lenselink^1‡^, Thijs Beuming^2^, Corine van Veen^1^, Arnault Massink^1^, Woody Sherman^2^, Herman W. T van Vlijmen^1^, Adriaan P. IJzerman^1*^

1. Division of Medicinal Chemistry, Leiden Academic Centre for Drug Research, Leiden University, Leiden, The Netherlands

*2. Schrödinger, Inc., 120 West 45th Street, New York, New York 10036, United States*

Corresponding Author: Prof. Dr. Adriaan P IJzerman, Leiden Academic Center for Drug Research, Leiden University, Einsteinweg 55, 2333 CC Leiden, the Netherlands

Phone: +31 (0)71 527 4651, Fax; +31 (0)71 527 4277

E-mail: [ijzerman@lacdr.leidenuniv.nl](mailto:ijzerman@lacdr.leidenuniv.nl)

Table 1: Displacement of the 73 non active molecules page 3-16

Table 2: Displacement of 8 additional non active molecules page 16,17

Figure 3: Docking pose of the two active compounds page 18

Figure 4: Docking pose of five inactive compounds page 19-21

Figure 3: HPLC spectrum of the fist active compound page 22

Figure 4: HPLC spectrum of second active compound page 23

Figure 5: ^1^H NMR spectrum of the first active compound page 24

Figure 6: ^1^H NMR spectrum of the second active compound page 25

SI table 1. % Displacement of [^3^H]ZM-241385 binding from the adenosine A_2A_ receptor by the 69 non-active compounds on the adenosine A_2A_ receptor. Docking score and rank are given of the first node of the decision tree (figure 2).

| Structure | Displacement at 10 μM (%) | Docking score (kcal/mol)  Rank | Smiles |
| --- | --- | --- | --- |
| 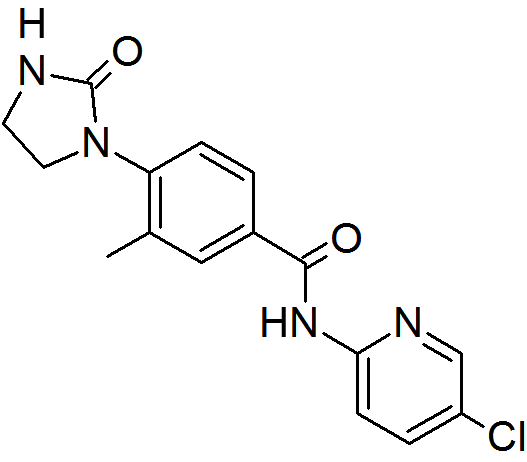 | 26 ± 2 % | -9.01  8,498 | n1cc(Cl)ccc1NC(=O)c(cc2C)ccc2N3CCNC3=O |
| 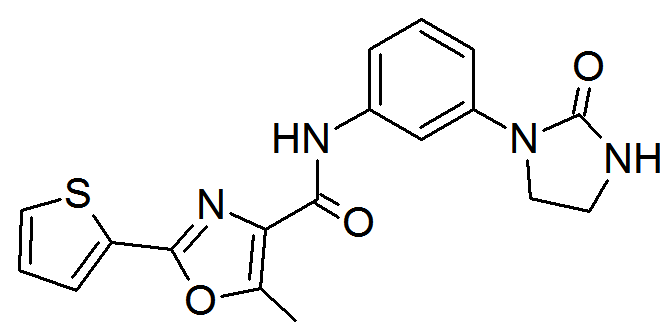 | 24 ± 2 % | -9.64  1,291 | c1ccsc1-c2oc(C)c(n2)C(=O)Nc3cccc(c3)N4CCNC4=O |
| 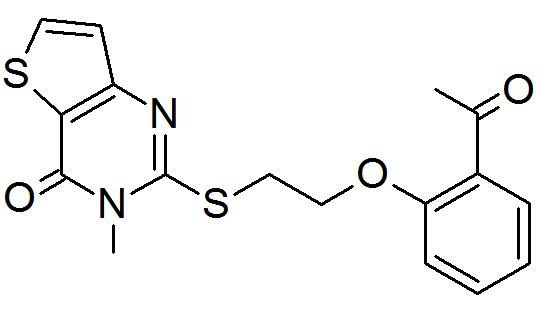 | 21 ± 1 % | -6.58  976,740 | CC(=O)c1ccccc1OCCSc(n2)n(C)c(=O)c(c23)scc3 |
| 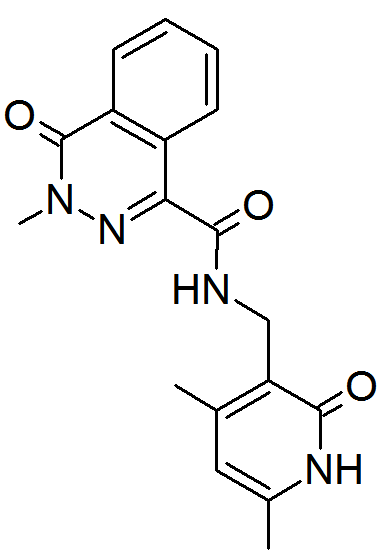 | 20 ± 4 % | -6.28  1,359,475 | Cc1cc(C)c(c(=O)[nH]1)CNC(=O)c(nn(C)c2=O)c(c23)cccc3 |
| 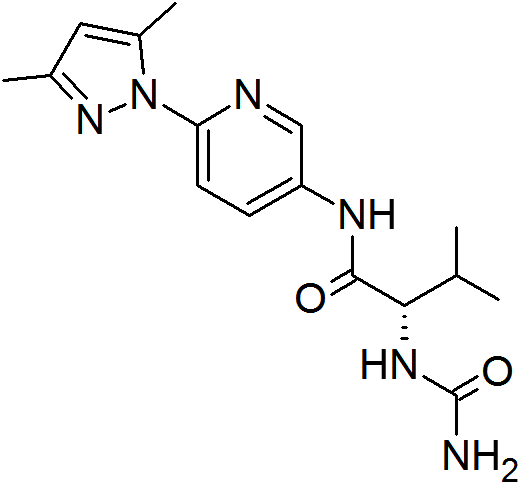 | 19 ± 2 % | -6.42  1,174,461 | O=C(N)NC(C(C)C)C(=O)Nc1ccc(nc1)-n2nc(C)cc2C |
| 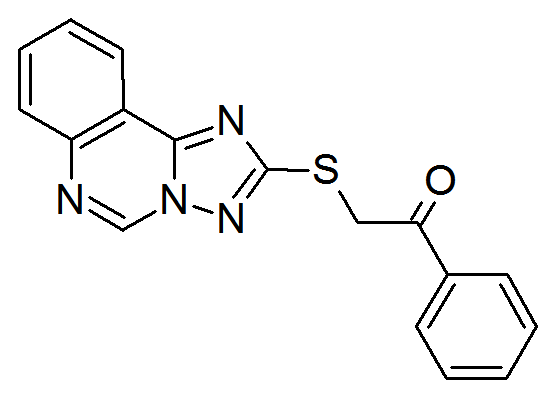 | 18 ± 2 % | -6.29  1,349,985 | c1ccccc1C(=O)CSc(n2)n3cnc(c4c23)cccc4 |
| 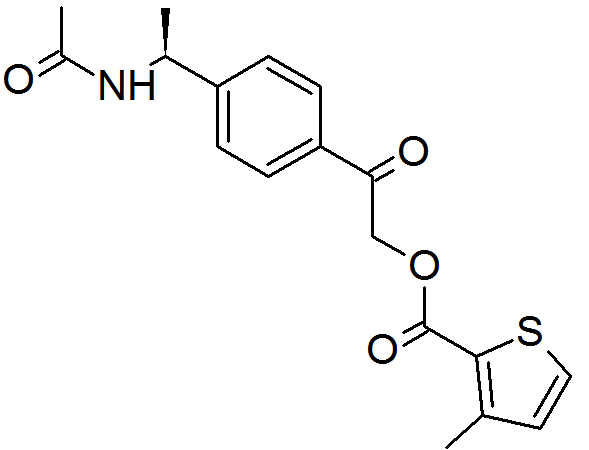 | 16 ± 2 % | -8.88  11,791 | O=C(C)NC(C)c1ccc(cc1)C(=O)COC(=O)c2sccc2C |
| 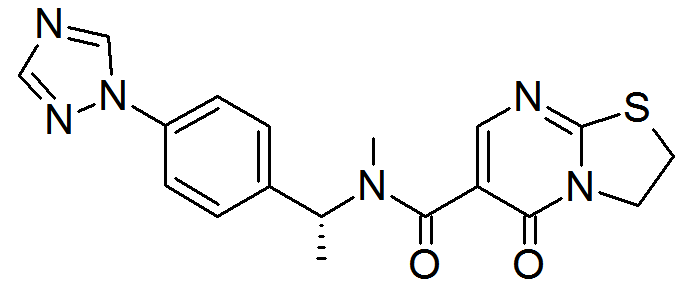 | 15 ± 3 % | -8.38  41,382 | S1CCn(c12)c(=O)c(cn2)C(=O)N(C)C(C)c3ccc(cc3)-n4cncn4 |
| 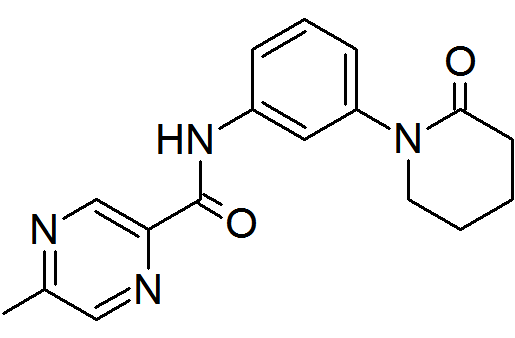 | 14 ± 3 % | -8.93  10,481 | Cc1cnc(cn1)C(=O)Nc2cccc(c2)N3CCCCC3=O |
| 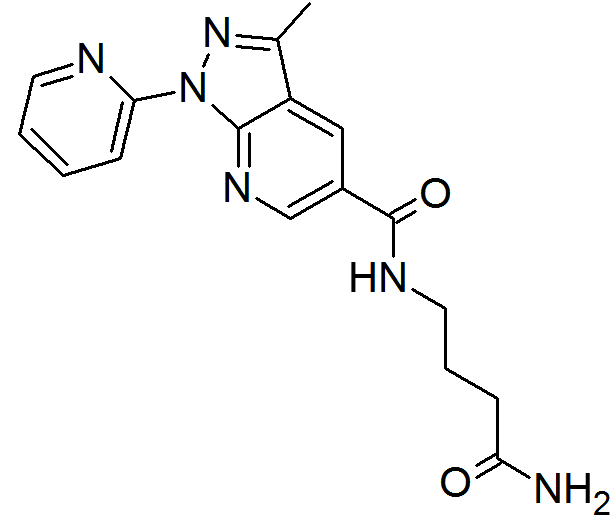 | 13 ± 2 % | -8.92  10,739 | n1ccccc1-n2nc(C)c(c23)cc(cn3)C(=O)NCCCC(=O)N |
| 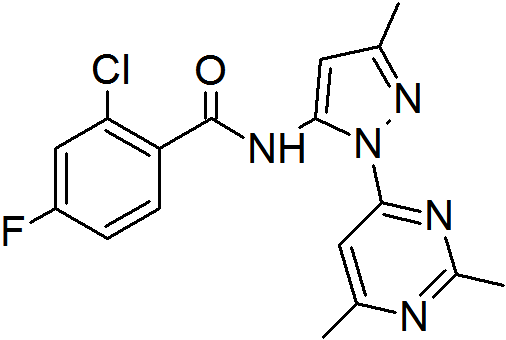 | 13 ± 3 % | -9.28  3,890 | Cc(n1)nc(C)cc1-n(n2)c(cc2C)NC(=O)c3ccc(F)cc3Cl |
| 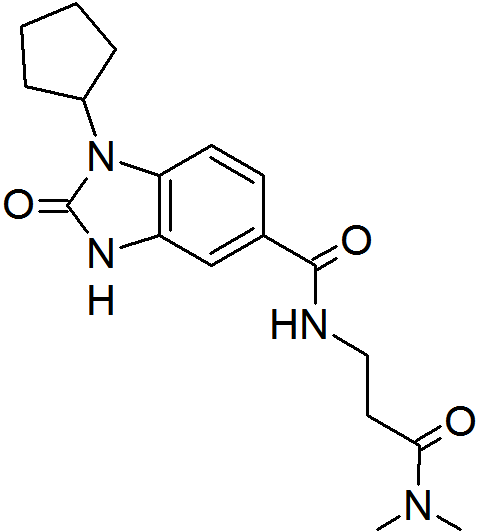 | 13 ± 6 % | -6.61  945,052 | CN(C)C(=O)CCNC(=O)c(cc1)cc(c12)[nH]c(=O)n2C3CCCC3 |
| 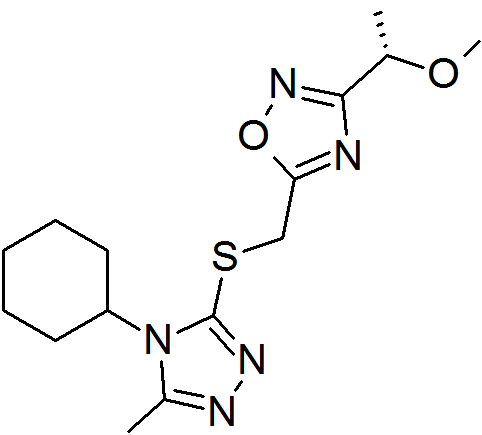 | 12 ± 5 % | -9.03  8,071 | COC(C)c1noc(n1)CSc2nnc(C)n2C3CCCCC3 |
| 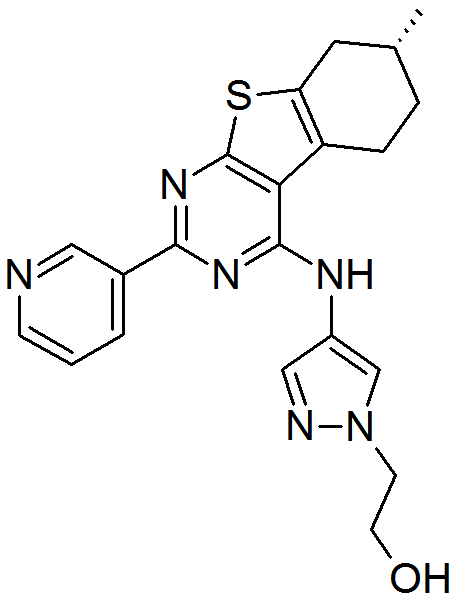 | 11 ± 2 % | -9.08  6,867 | C1CC(C)Cc(c12)sc3c2c(nc(n3)-c4cccnc4)Nc5cnn(c5)CCO |
| 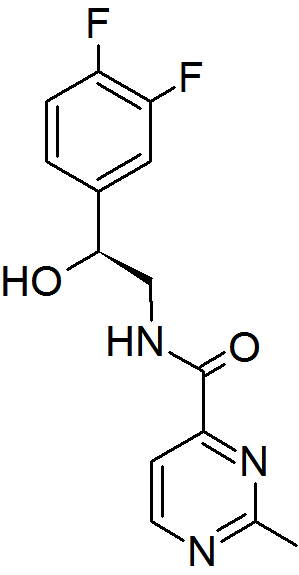 | 8 ± 1 % | -9.20  4,939 | Cc1nccc(n1)C(=O)NCC(O)c2ccc(F)c(F)c2 |
| 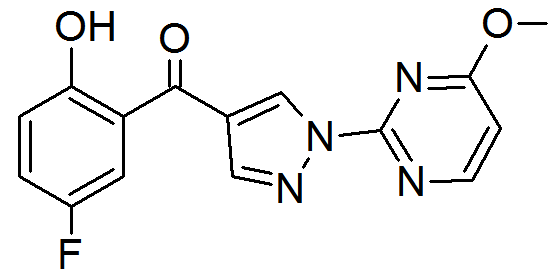 | 8 ± 4 % | -9.26  4,184 | COc1ccnc(n1)-n2cc(cn2)C(=O)c3cc(F)ccc3O |
| 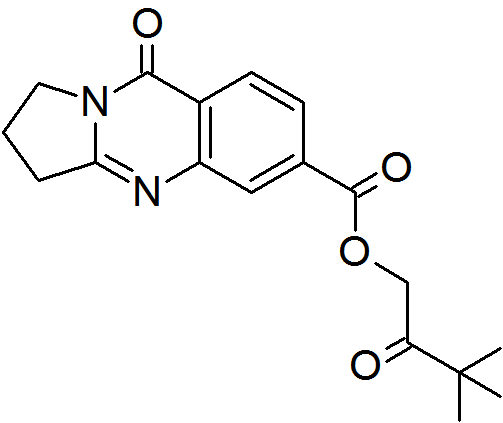 | 8 ± 4 % | -4.12  4,244,394 | CC(C)(C)C(=O)COC(=O)c(cc1)cc(c12)nc3n(c2=O)CCC3 |
| 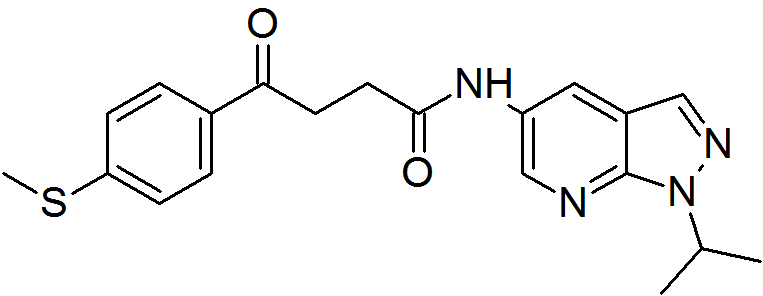 | 8 ± 4 % | -7.91  116,821 | CSc1ccc(cc1)C(=O)CCC(=O)Nc(cn2)cc(c23)cnn3C(C)C |
| 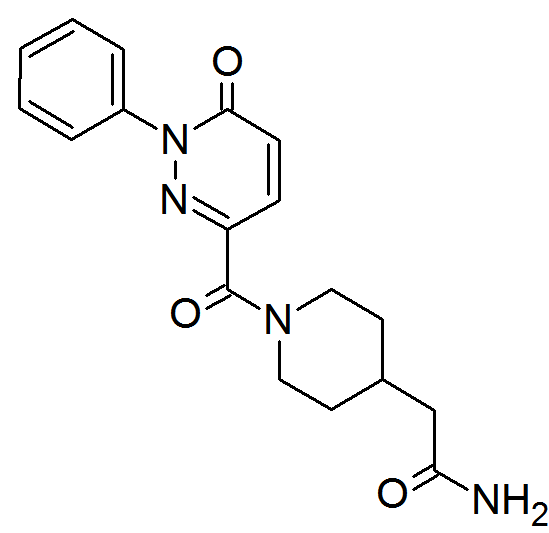 | 8 ± 5 % | -7.20  256,227 | NC(=O)CC1CCN(CC1)C(=O)c2ccc(=O)n(n2)-c3ccccc3 |
| 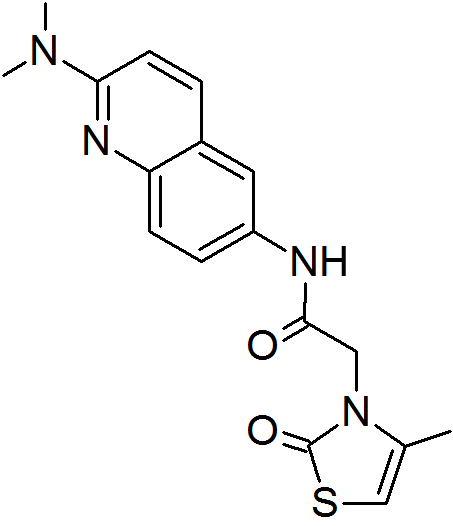 | 7 ± 4 % | -7.11  478,261 | CN(C)c(cc1)nc(c12)ccc(c2)NC(=O)Cn3c(C)csc3=O |
| 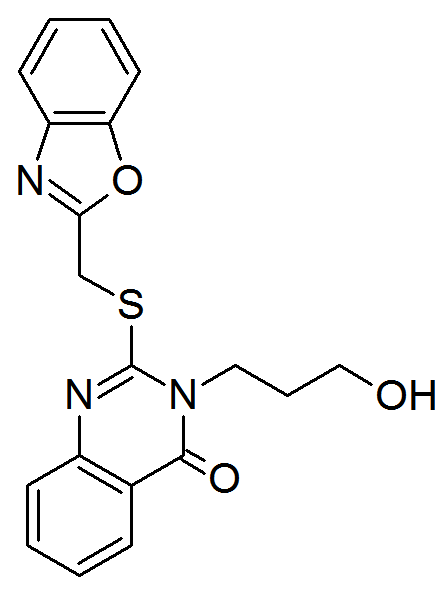 | 7 ± 3 % | -9.65  1,258 | c1cccc(c12)c(=O)n(CCCO)c(n2)SCc(n3)oc(c34)cccc4 |
| 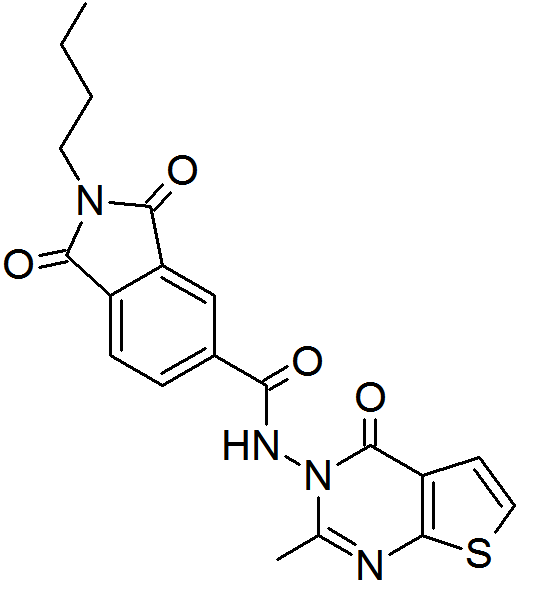 | 6 ± 6 % | -6.58  975.625 | CCCCN(C1=O)C(=O)c(c12)cc(cc2)C(=O)Nn(c(n3)C)c(=O)c(c34)ccs4 |
| 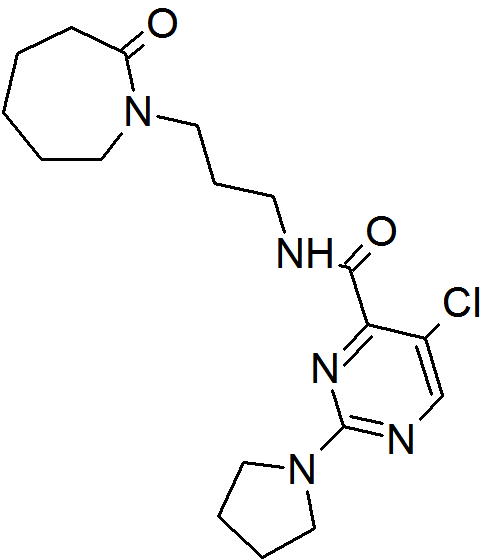 | 6 ± 2 % | -9.48  2,101 | C1CCCN1c(ncc2Cl)nc2C(=O)NCCCN3CCCCCC3=O |
| 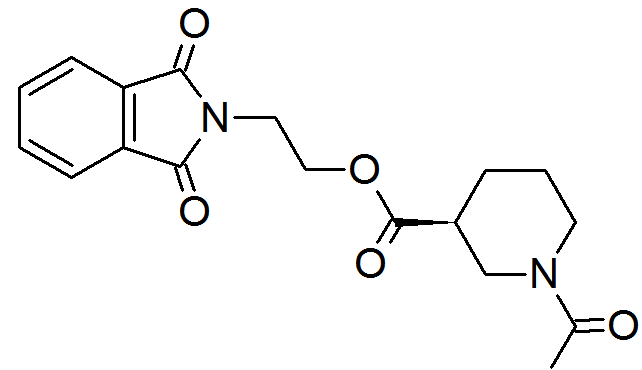 | 6 ± 5 % | -7.77  153,617 | CC(=O)N1CCCC(C1)C(=O)OCCN(C2=O)C(=O)c(c23)cccc3 |
| 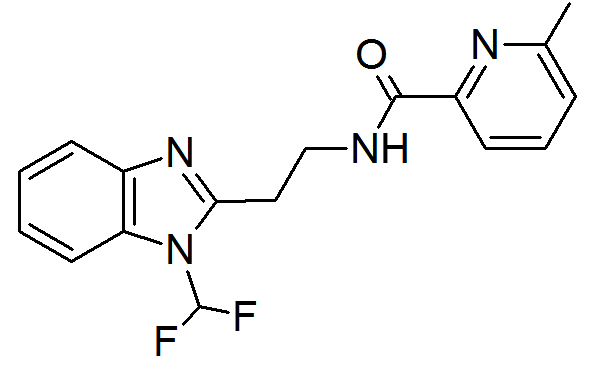 | 6 ± 2 % | -9.22  4,621 | Cc1cccc(n1)C(=O)NCCc(n2)n(C(F)F)c(c23)cccc3 |
| 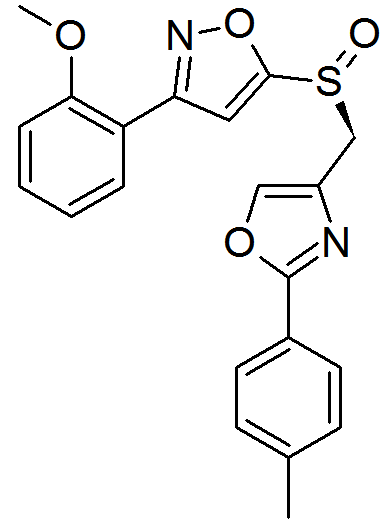 | 6 ± 1 % | -9.77  831 | COc1ccccc1-c2cc(on2)CS(=O)Cc3coc(n3)-c4ccc(C)cc4 |
| 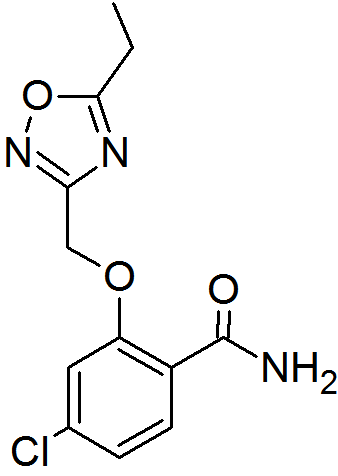 | 5 ± 2 % | -8.98  9,229 | CCc1nc(no1)COc2cc(Cl)ccc2C(=O)N |
| 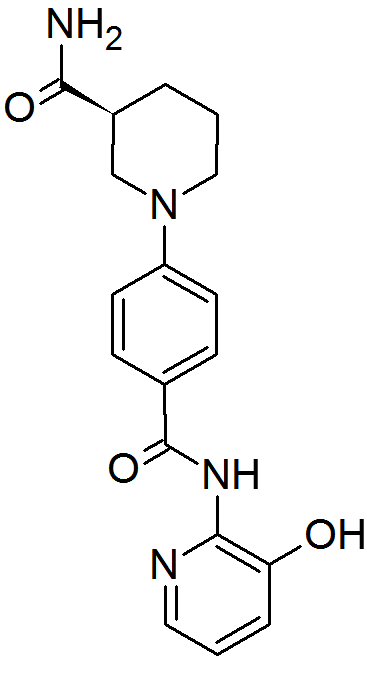 | 5 ± 3 % | -9.60  1,467 | NC(=O)C1CCCN(C1)c2ccc(cc2)C(=O)Nc3ncccc3O |
| 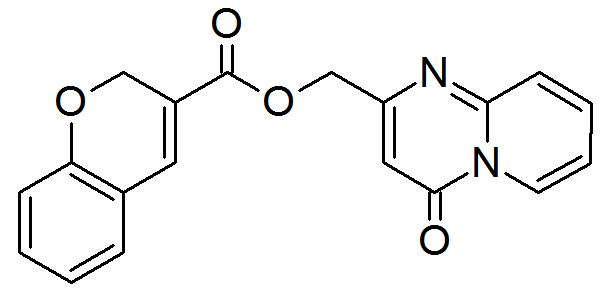 | 4 ± 4 % | -6.61  944.201 | c1cccc(c12)OCC(=C2)C(=O)OCc(cc3=O)nc(n34)cccc4 |
| 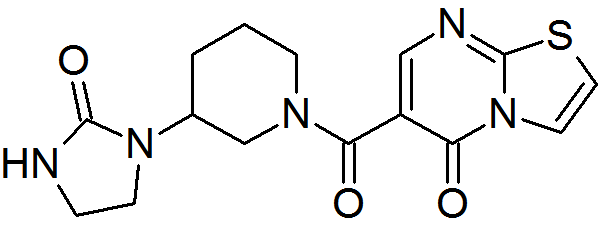 | 4 ± 1 % | -9.23  4465 | s1ccn(c12)c(=O)c(cn2)C(=O)N3CCCC(C3)N4CCNC4=O |
| 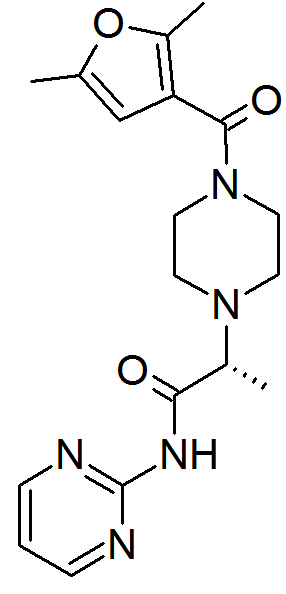 | 4 ± 3 % | -9.28  3,953 | n1cccnc1NC(=O)C(C)N2CCN(CC2)C(=O)c3cc(C)oc3C |
| 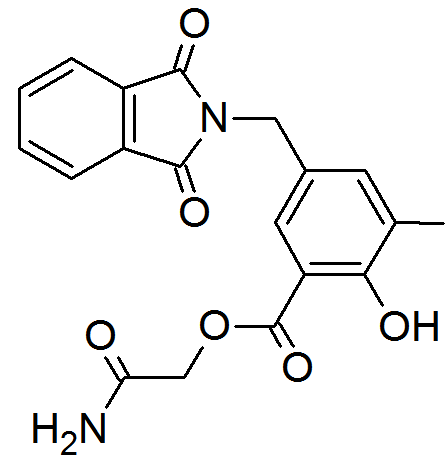 | 3 ± 1 % | -9.46  2,292 | c1cccc(c12)C(=O)C(C2=O)Cc3cc(c(O)c(c3)C)C(=O)OCC(=O)N |
| 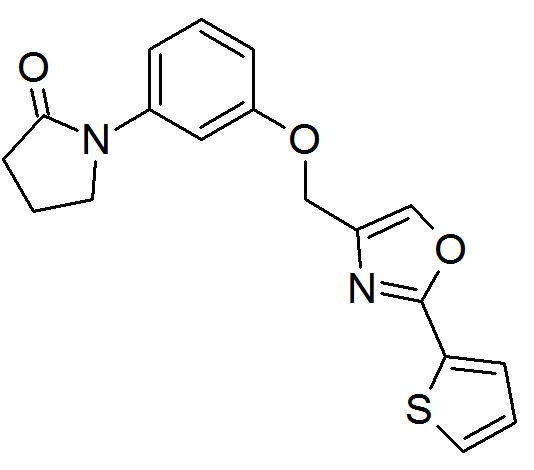 | 3 ± 3 % | -9.48  2,090 | C1CCC(=O)N1c(ccc2)cc2OCc3nc(oc3)-c4sccc4 |
| 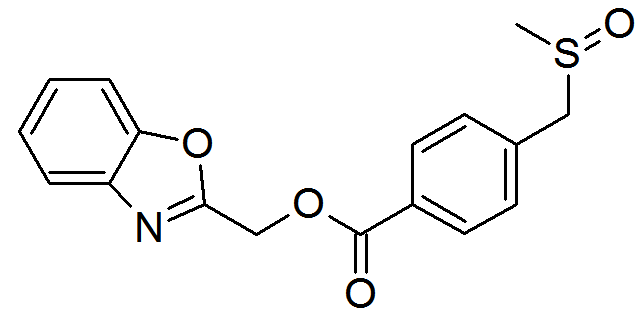 | 3 ± 2 % | -9.45  2349 | c1cccc(c12)oc(n2)COC(=O)c3ccc(cc3)CS(=O)C |
| 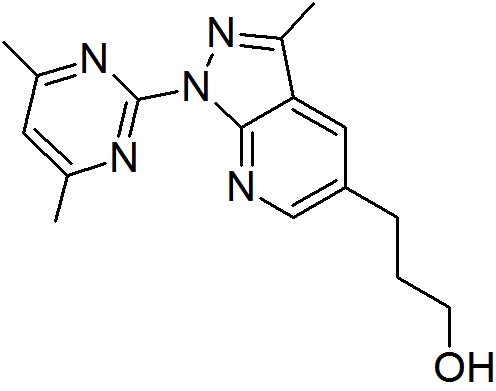 | 3 ± 2 % | -9.08  6,870 | OCCCc(cn1)cc(c12)c(C)nn2-c3nc(C)cc(n3)C |
| 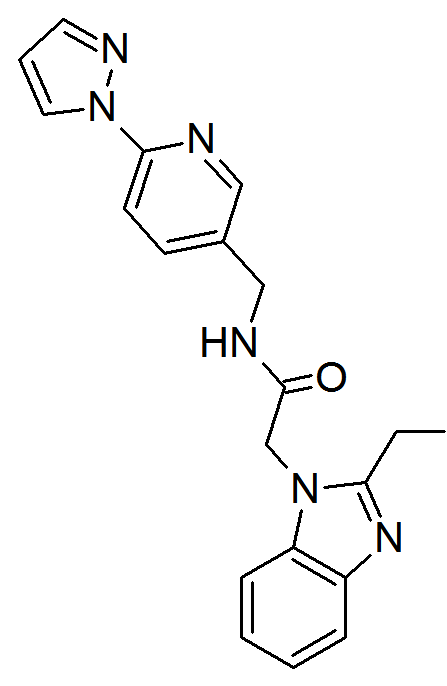 | 2 ± 2 % | -9.44  2,417 | c1cccc(c12)n(c(n2)CC)CC(=O)NCc3ccc(nc3)-n4cccn4 |
| 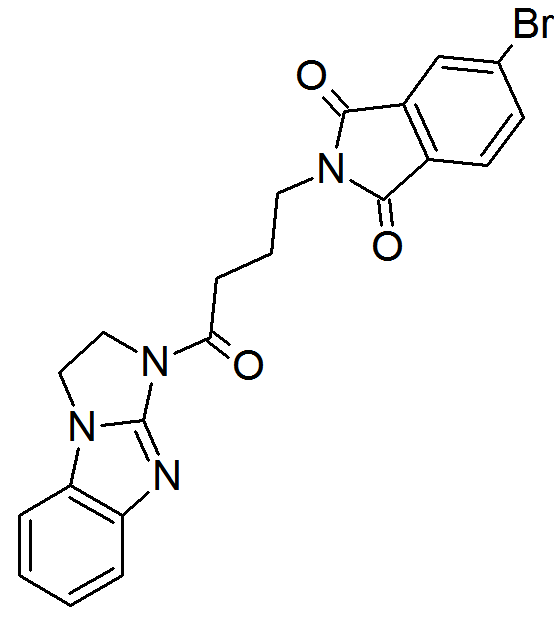 | 2 ± 1 % | -9.91  528 | c1cc(Br)cc(c12)C(=O)N(C2=O)CCCC(=O)N(CC3)c(n34)nc5c4cccc5 |
| 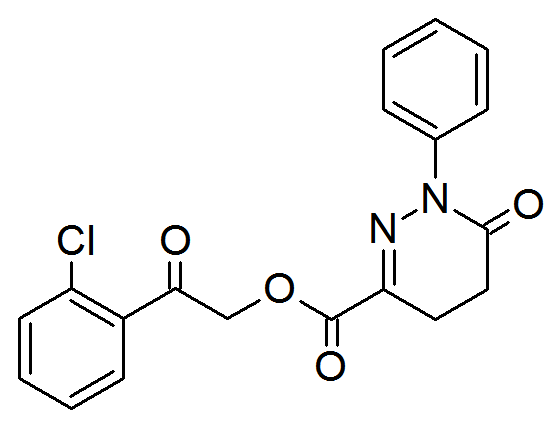 | 2 ± 2 % | -8.16  68.635 | Clc1ccccc1C(=O)COC(=O)C2=NN(C(=O)CC2)c3ccccc3 |
| 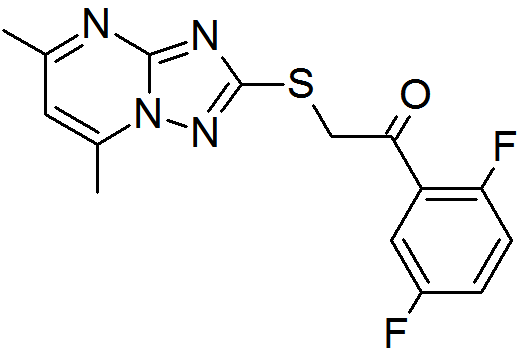 | 2 ± 2 % | -9.40  2,786 | Cc1cc(C)nc(n12)nc(n2)SCC(=O)c3cc(F)ccc3F |
| 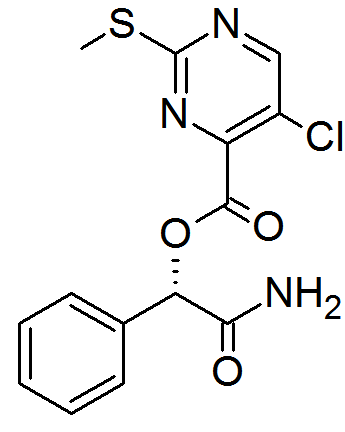 | 1 ± 3 % | -9.14  5,762 | CSc1ncc(Cl)c(n1)C(=O)OC(C(=O)N)c2ccccc2 |
| 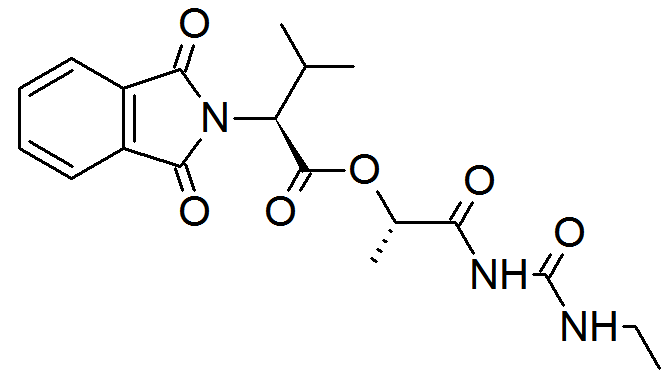 | 1 ± 3 % | -8.80  14,726 | CCNC(=O)NC(=O)C(C)OC(=O)C(C(C)C)N(C1=O)C(=O)c(c12)cccc2 |
| 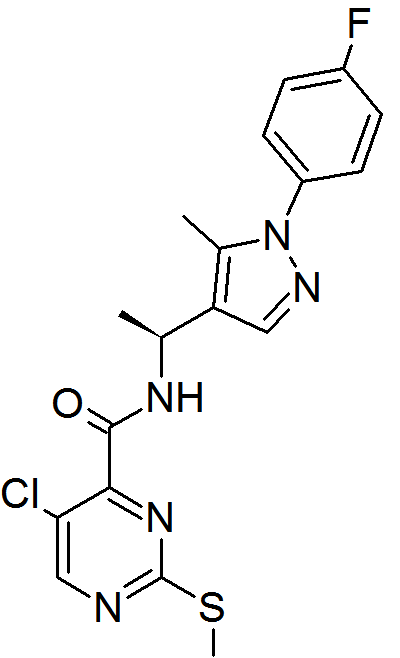 | 1 ± 3 % | -9.98  406 | CSc1ncc(Cl)c(n1)C(=O)NC(C)c2cnn(c2C)-c3ccc(F)cc3 |
| 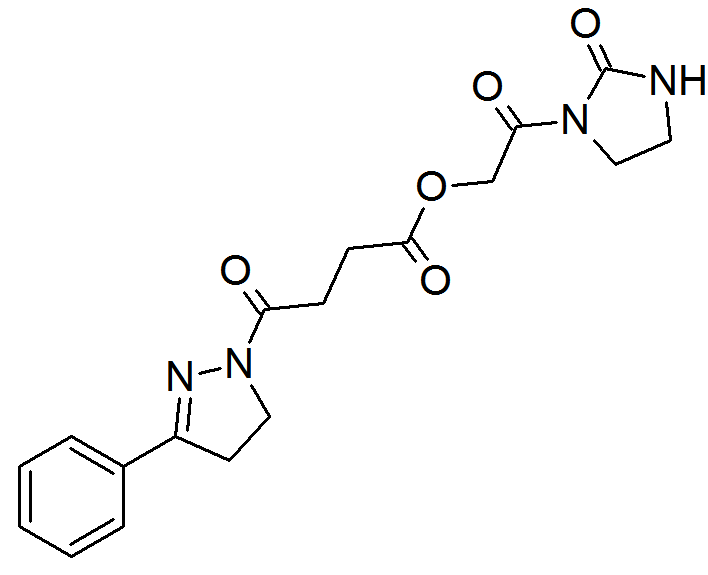 | 1 ± 3 % | -8.96  9,712 | O=C1NCCN1C(=O)COC(=O)CCC(=O)N2CCC(=N2)c3ccccc3 |
| 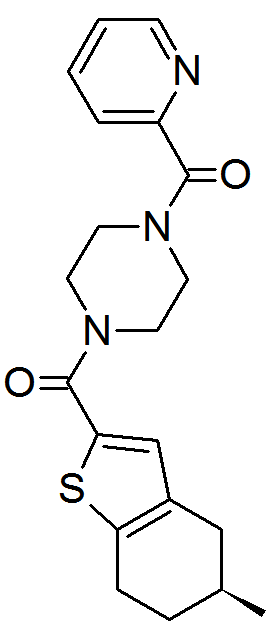 | 1 ± 2 % | -9.17  5,320 | C1CC(C)Cc(c12)cc(s2)C(=O)N3CCN(CC3)C(=O)c4ccccn4 |
| 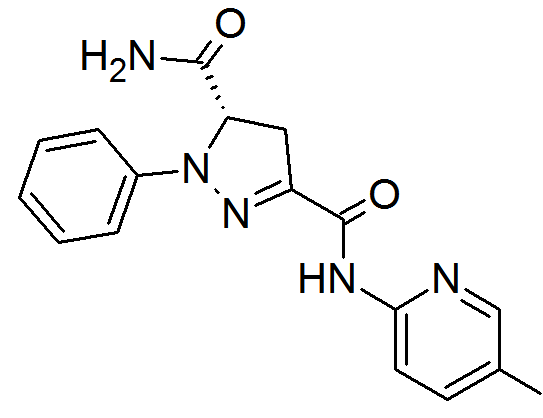 | 0 ± 2 % | -8.95  10,012 | Cc1ccc(nc1)NC(=O)C2=NN(C(C2)C(=O)N)c3ccccc3 |
| 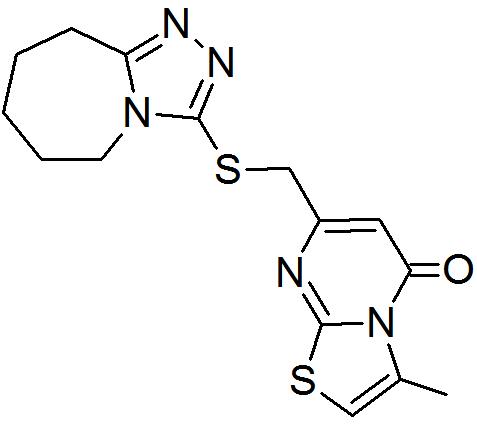 | 0 ± 4 % | -7.02  545,507 | s1cc(C)n(c12)c(=O)cc(n2)CSc(nn3)n(c34)CCCCC4 |
| 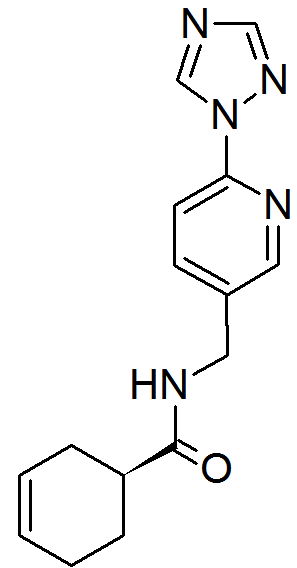 | 0 ± 2 % | -9.81  741 | C1C=CCCC1C(=O)NCc2ccc(nc2)-n3cncn3 |
| 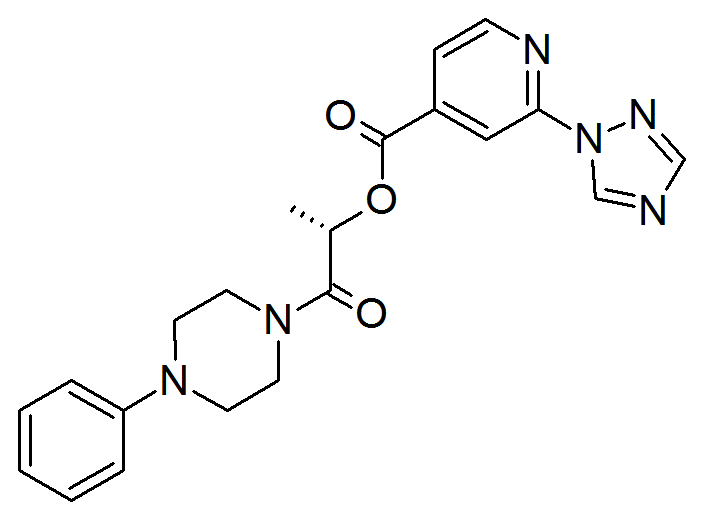 | 0 ± 3 % | -8.63  22,708 | c1ccccc1N(CC2)CCN2C(=O)C(C)OC(=O)c3ccnc(c3)-n4cncn4 |
| 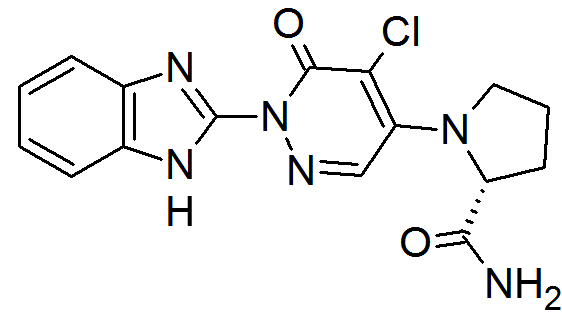 | 0 ± 3 % | -10.00  370 | NC(=O)C1CCCN1c2cnn(c(=O)c2Cl)-c(n3)[nH]c(c34)cccc4 |
| 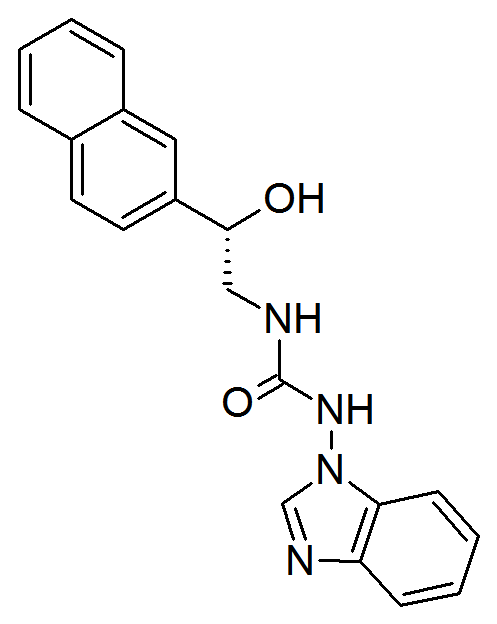 | -2 ± 3 % | -5.75  2,167,687 | c1cccc(c12)cc(cc2)C(O)CNC(=O)Nn(cn3)c(c34)cccc4 |
| 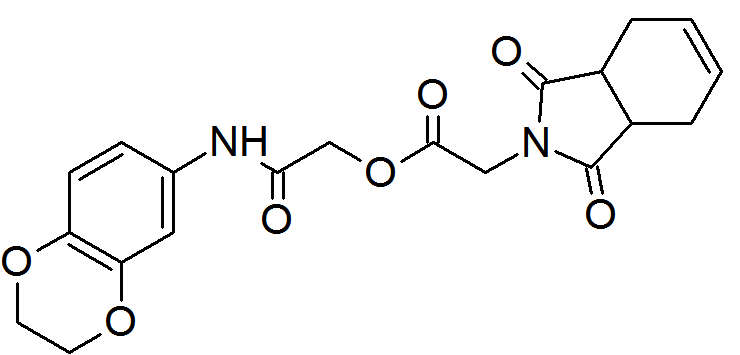 | -2 ± 2 % | -9.00  8,334 | O1CCOc(c12)cc(cc2)NC(=O)COC(=O)CN(C3=O)C(=O)C(C34)CC=CC4 |
| 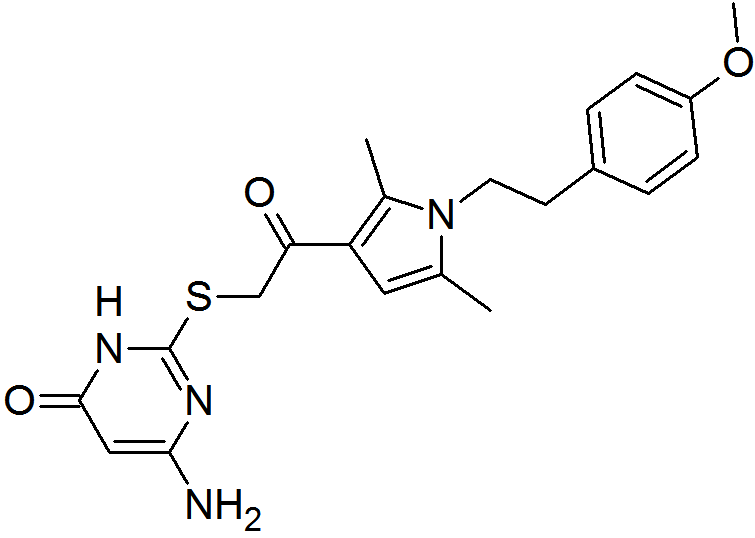 | -4 ± 3 % | -9.33  3,370 | COc1ccc(cc1)CCn2c(C)cc(c2C)C(=O)CSc3nc(N)cc(=O)[nH]3 |
| 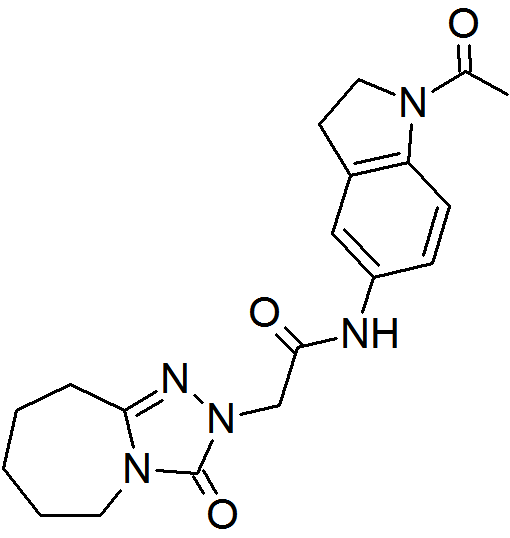 | -6 ± 2 % | -7.65  192,364 | CC(=O)N(CC1)c(c12)ccc(c2)NC(=O)Cn(n3)c(=O)n(c34)CCCCC4 |
| 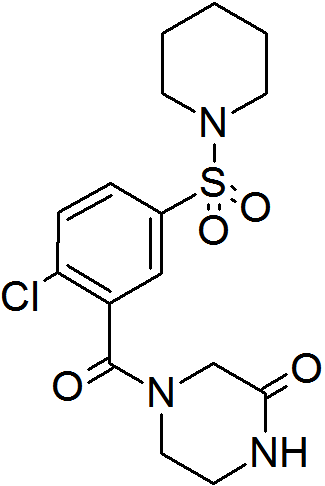 | -7 ± 2 % | -9.04  7,632 | C1CCCCN1S(=O)(=O)c(ccc2Cl)cc2C(=O)N3CCNC(=O)C3 |
| 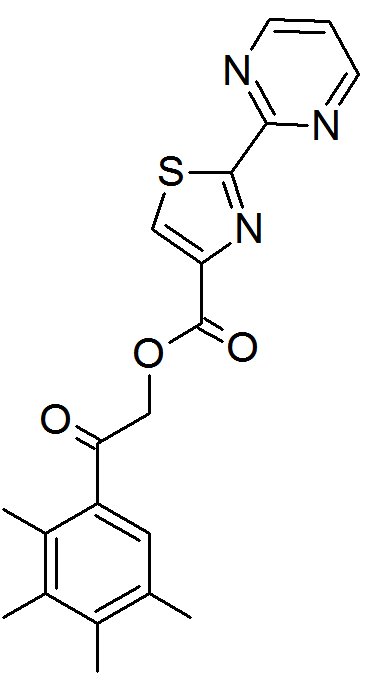 | -7 ± 2 % | -9.54  1,786 | Cc1c(C)c(C)c(cc1C)C(=O)COC(=O)c2csc(n2)-c3ncccn3 |
| 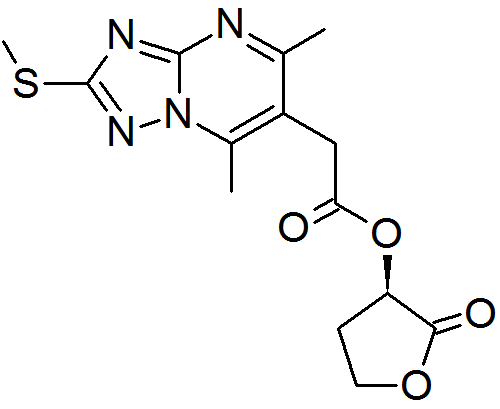 | -8 ± 2 % | -8.90  11,398 | CSc(n1)nn(c12)c(C)c(c(n2)C)CC(=O)OC3CCOC3=O |
| 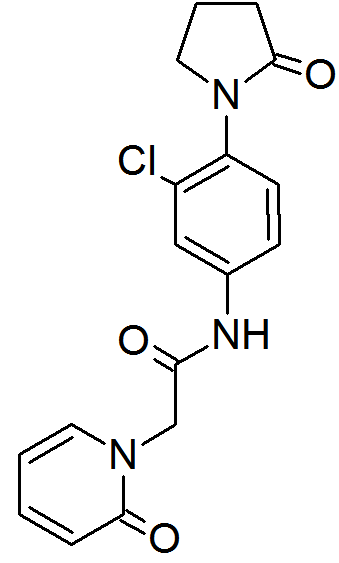 | -8 ± 1 % | -9.42  2,579 | O=C1CCCN1c2ccc(cc2Cl)NC(=O)Cn3ccccc3=O |
| 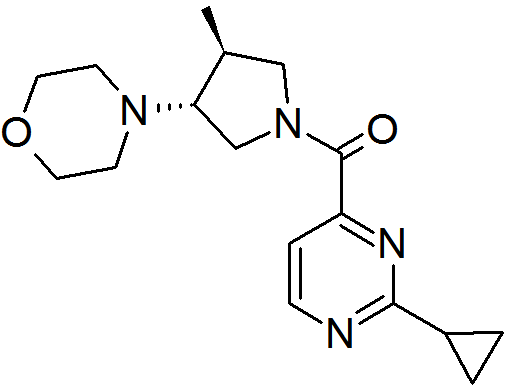 | -8 ± 4 % | -9.14  5,760 | C1COCCN1C2CN(CC2C)C(=O)c3ccnc(n3)C4CC4 |
| 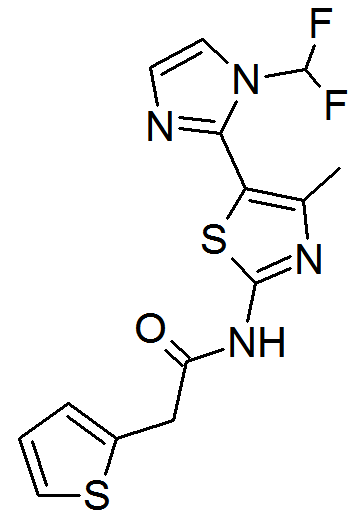 | -8 ± 5 % | -9.27  4,022 | s1cccc1CC(=O)Nc(nc2C)sc2-c3nccn3C(F)F |
| 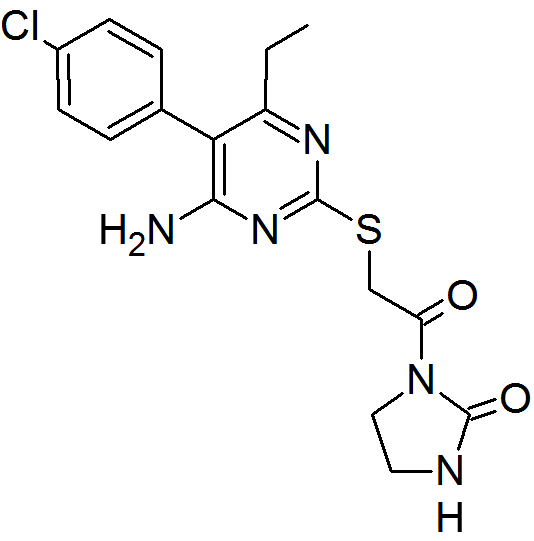 | -8 ± 6 % | -9.44  2,445 | c1cc(Cl)ccc1-c2c(N)nc(nc2CC)SCC(=O)N3CCNC3=O |
| 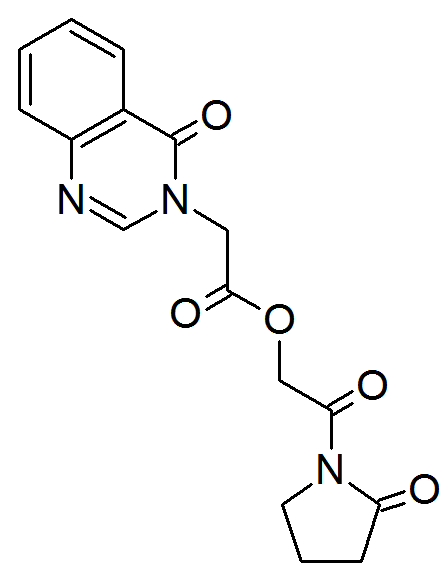 | -9 ± 3 % | -9.22  4,630 | c1cccc(c12)c(=O)n(cn2)CC(=O)OCC(=O)N3CCCC3=O |
| 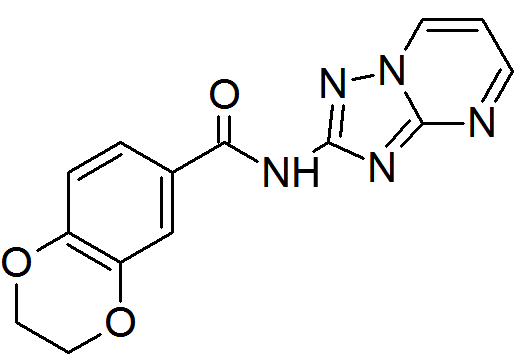 | -9 ± 2 % | -8.98  9,276 | O1CCOc(c12)cc(cc2)C(=O)Nc(n3)nn(c34)cccn4 |
| 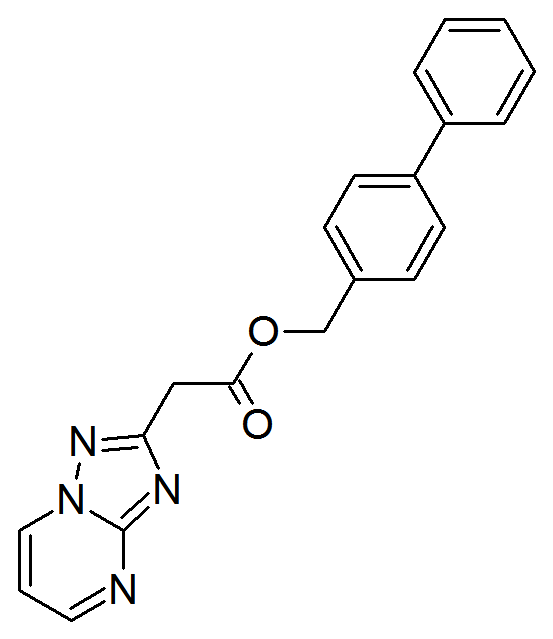 | -11 ± 2 % | -8.95  9,843 | n1cccn(c12)nc(n2)CC(=O)OCc3ccc(cc3)-c4ccccc4 |
| 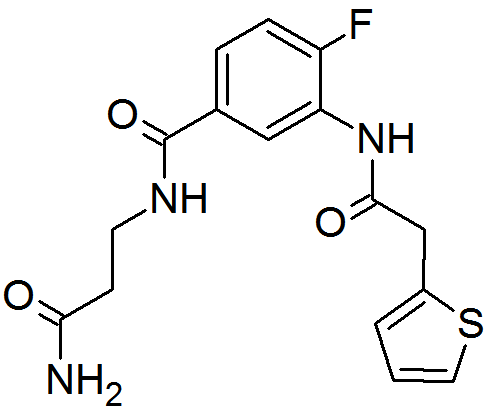 | -11 ± 4 % | -9.04  7,702 | NC(=O)CCNC(=O)c1ccc(F)c(c1)NC(=O)Cc2cccs2 |
| 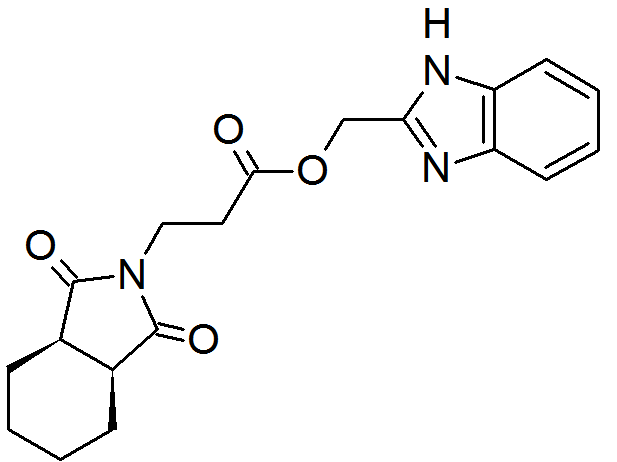 | -12 ± 2 % | -8.73  17,488 | c1cccc(c12)[nH]c(n2)COC(=O)CCN(C3=O)C(=O)C(C34)CCCC4 |
| 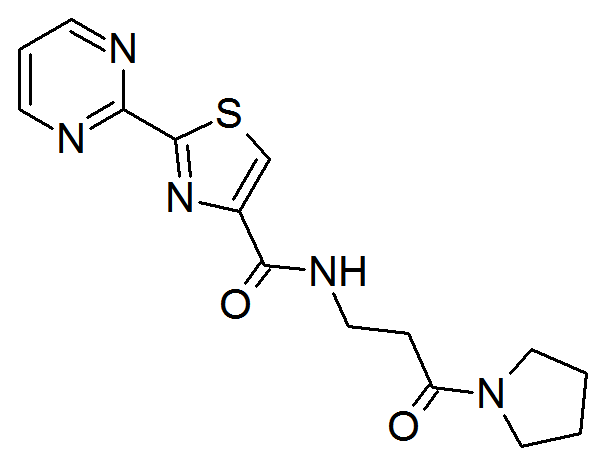 | -12 ± 6 % | -9.82  709 | C1CCCN1C(=O)CCNC(=O)c2csc(n2)-c3ncccn3 |
| 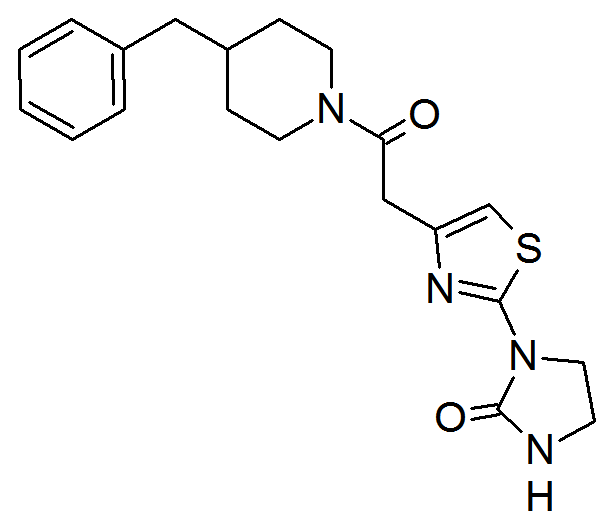 | -12 ± 4 % | -8.92  10,824 | c1ccccc1CC(CC2)CCN2C(=O)Cc3csc(n3)N4CCNC4=O |
| 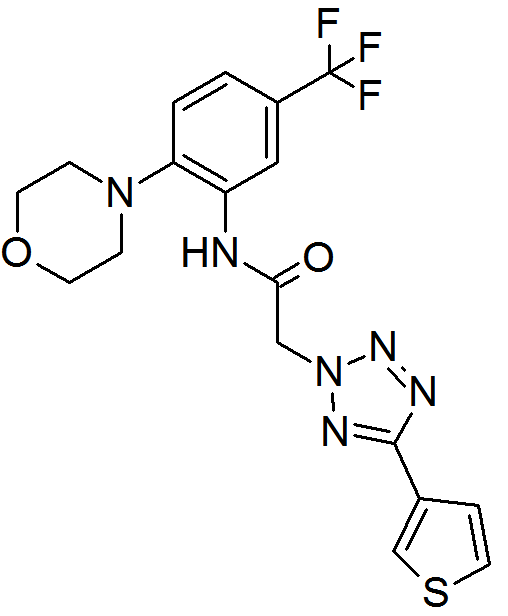 | -13 ± 4 % | -9.29  3,868 | C1COCCN1c(ccc2C(F)(F)F)c(c2)NC(=O)Cn3nnc(n3)-c4ccsc4 |
| 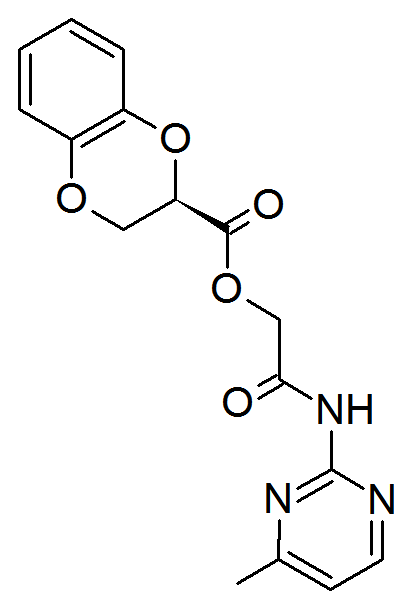 | -16 ± 3 % | -9.70  1,069 | Cc1ccnc(n1)NC(=O)COC(=O)C(CO2)Oc(c23)cccc3 |

SI table 2. The % displacement of [^3^H]ZM-241385 radioligand binding from the adenosine A_2A_ receptor by 8 compounds that were selected based on a bidentate interaction with Asn253^6.55^. Docking score and rank are given of the first node of the decision tree (figure 2).

| 2D Structure | Displacement (%) | Docking score (kcal/mol)  Rank | Smiles |
| --- | --- | --- | --- |
| 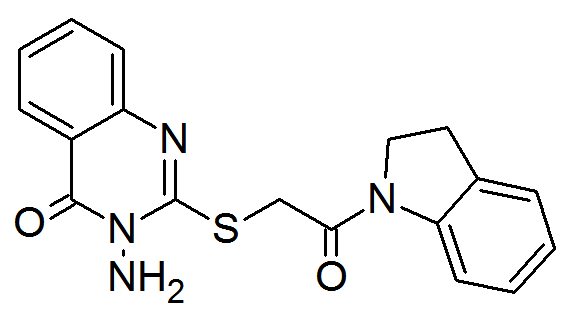 | 15 ± 1 % | -7.48  263,358 | c1cccc(c12)CCN2C(=O)CSc(n(N)c3=O)nc(c34)cccc4 |
| 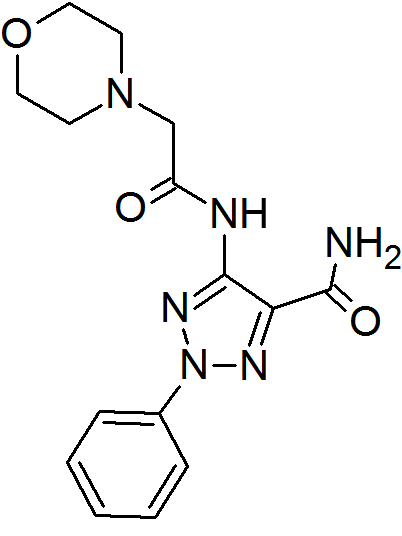 | 11 ± 3 % | -9.01  8,401 | C1COCCN1CC(=O)Nc2nn(nc2C(=O)N)-c3ccccc3 |
| 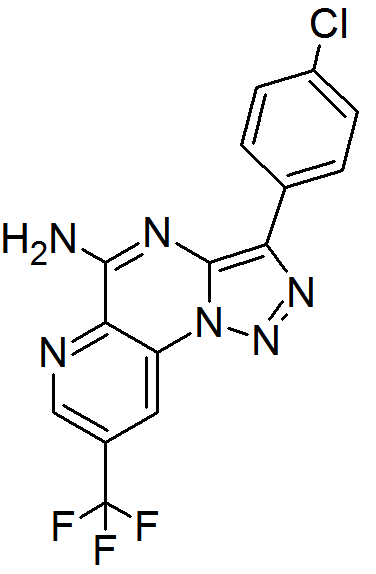 | 7 ± 3 % | -6.31  1,322,657 | Clc1ccc(cc1)-c2nnn(c23)c4c(c(n3)N)ncc(c4)C(F)(F)F |
| 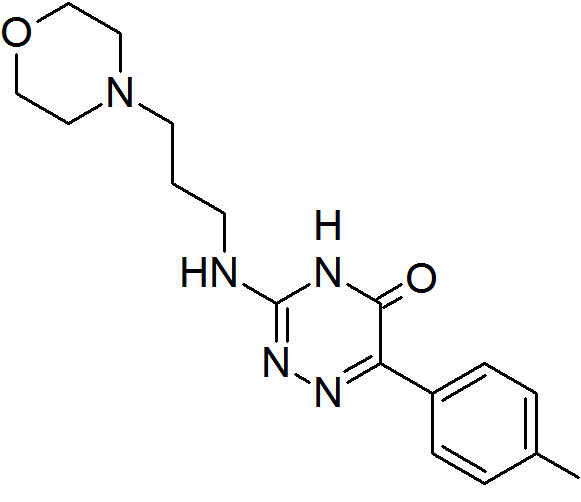 | 4 ± 3 % | -9.08  6,871 | Cc1ccc(cc1)-c2nnc([nH]c2=O)NCCCN3CCOCC3 |
| 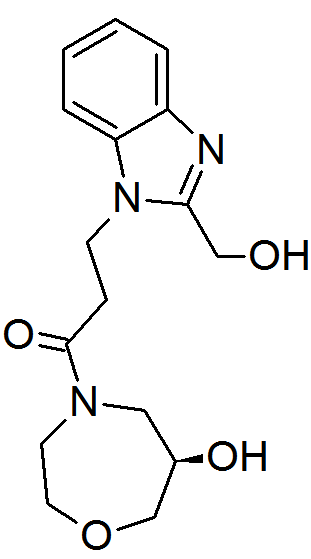 | 2 ± 3 % | -7.80  146,333 | c1cccc(c12)nc(CO)n2CCC(=O)N3CCOCC(O)C3 |
| 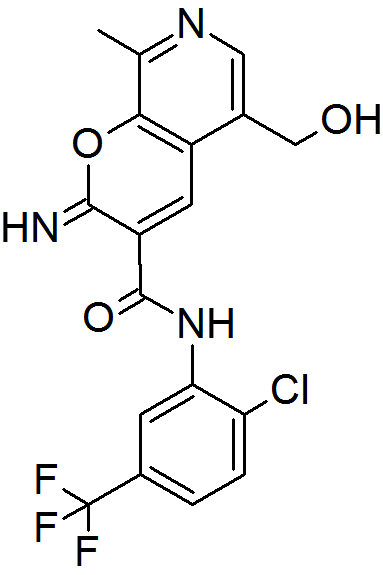 | 1 ± 4 % | -7.97  102,995 | OCc1cnc(C)c(c12)oc(=N)c(c2)C(=O)Nc3cc(C(F)(F)F)ccc3Cl |
| 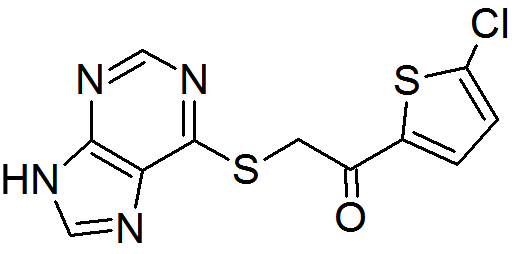 | -1 ± 2 % | -9.18  5,243 | Clc1ccc(s1)C(=O)CSc2ncnc(c23)[nH]cn3 |
| 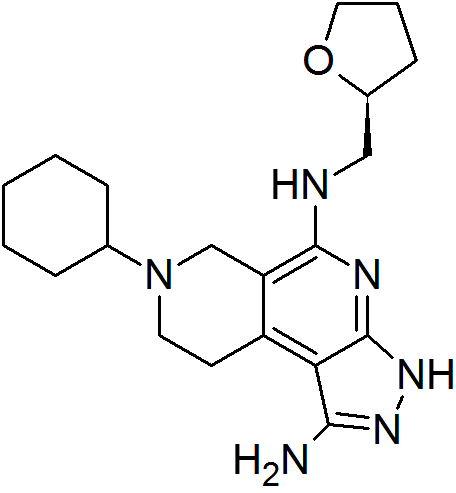 | -2 ± 3 % | -9.44  2,399 | C1CCCCC1N(C2)CCc(c23)c4c([nH]nc4N)nc3NCC5CCCO5 |

SI Figure 1, Docking poses of the two actives compounds in the adenosine A_2A_ receptor. Shown are residues involved in ligand binding. Both ligands interact with Asn253^6.55^


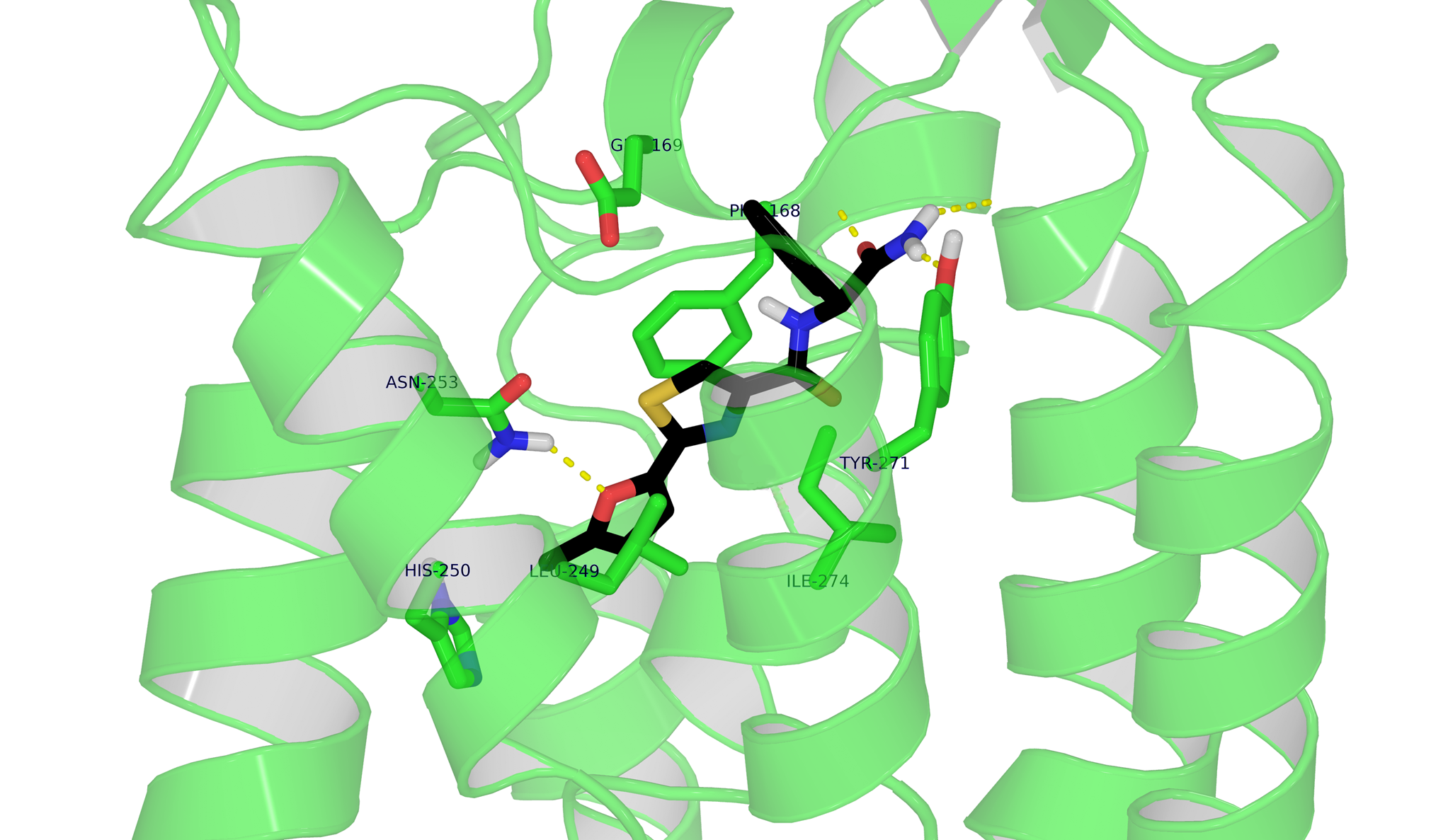

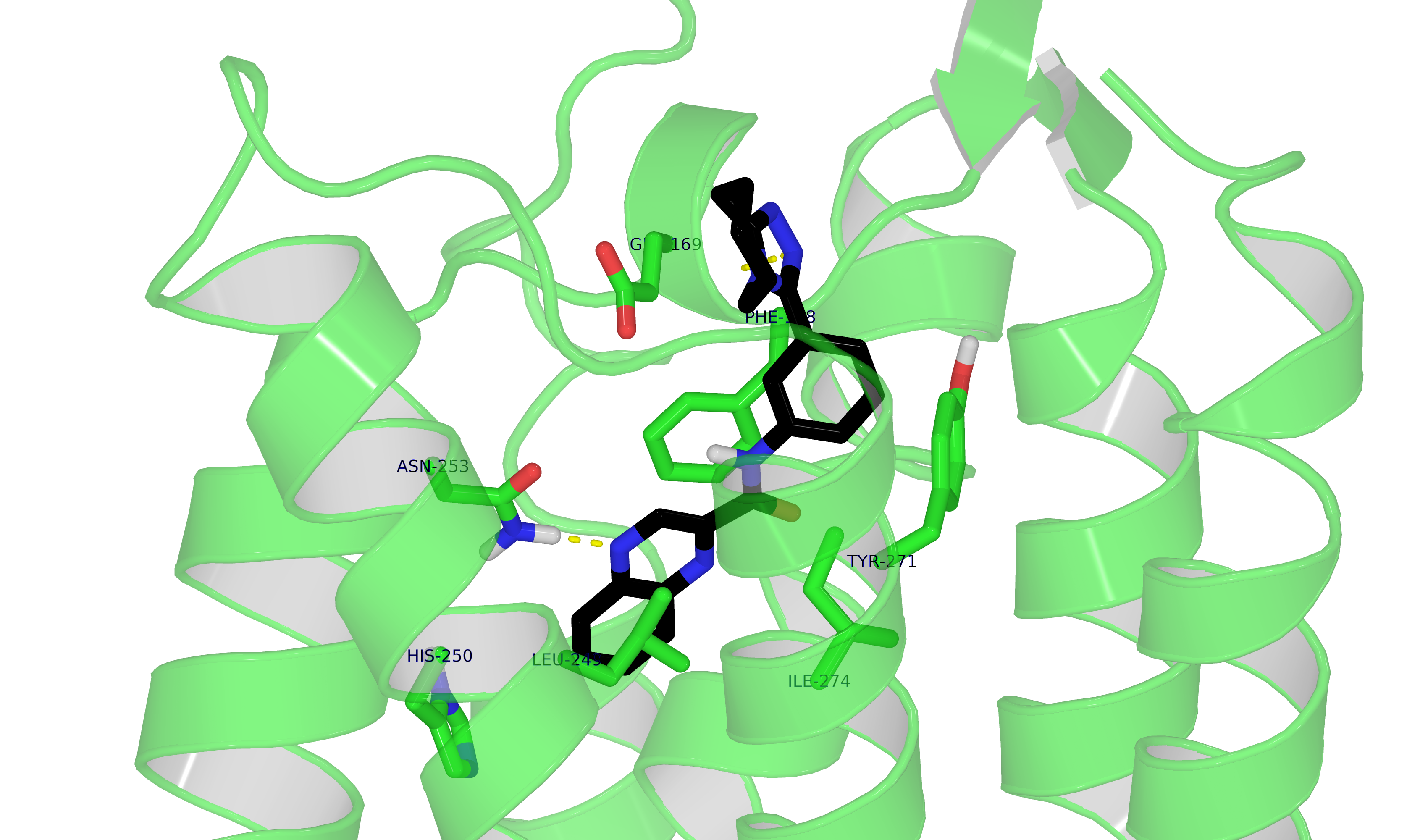


SI Figure 2, Docking poses of the top five highest scoring inactive compounds in the adenosine A_2A_ receptor. Shown are residues involved in ligand binding. All ligands interact with Asn253^6.55^.


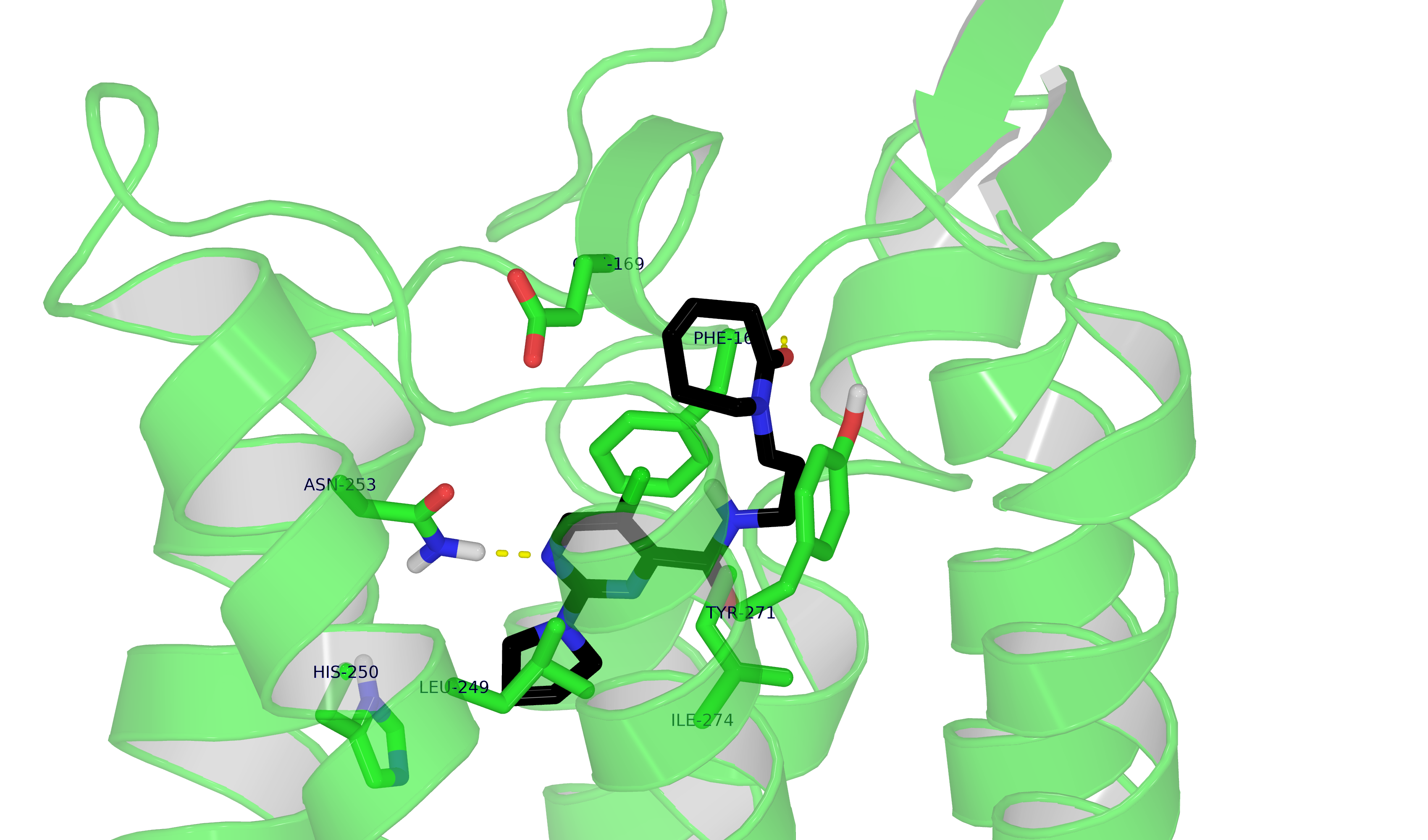

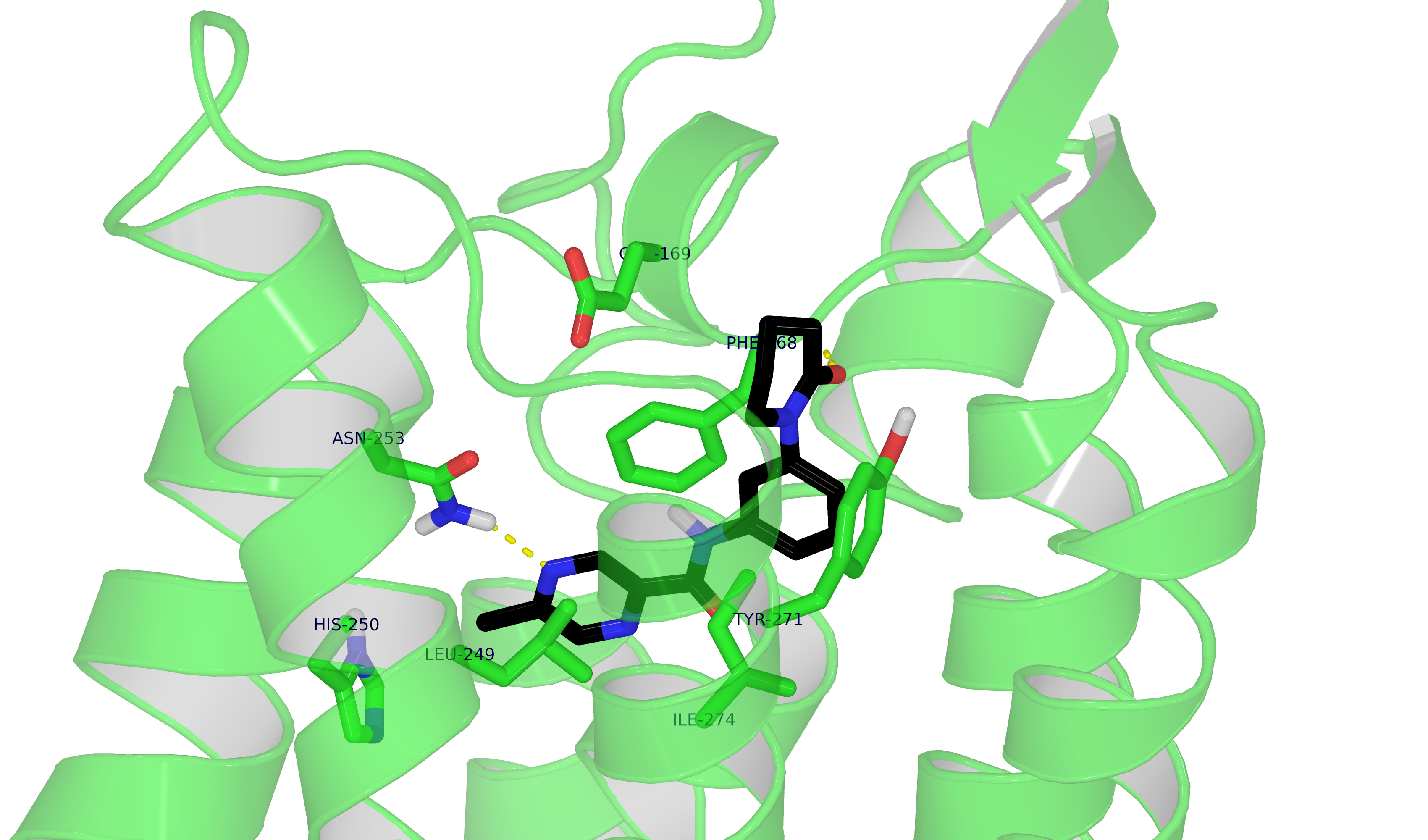


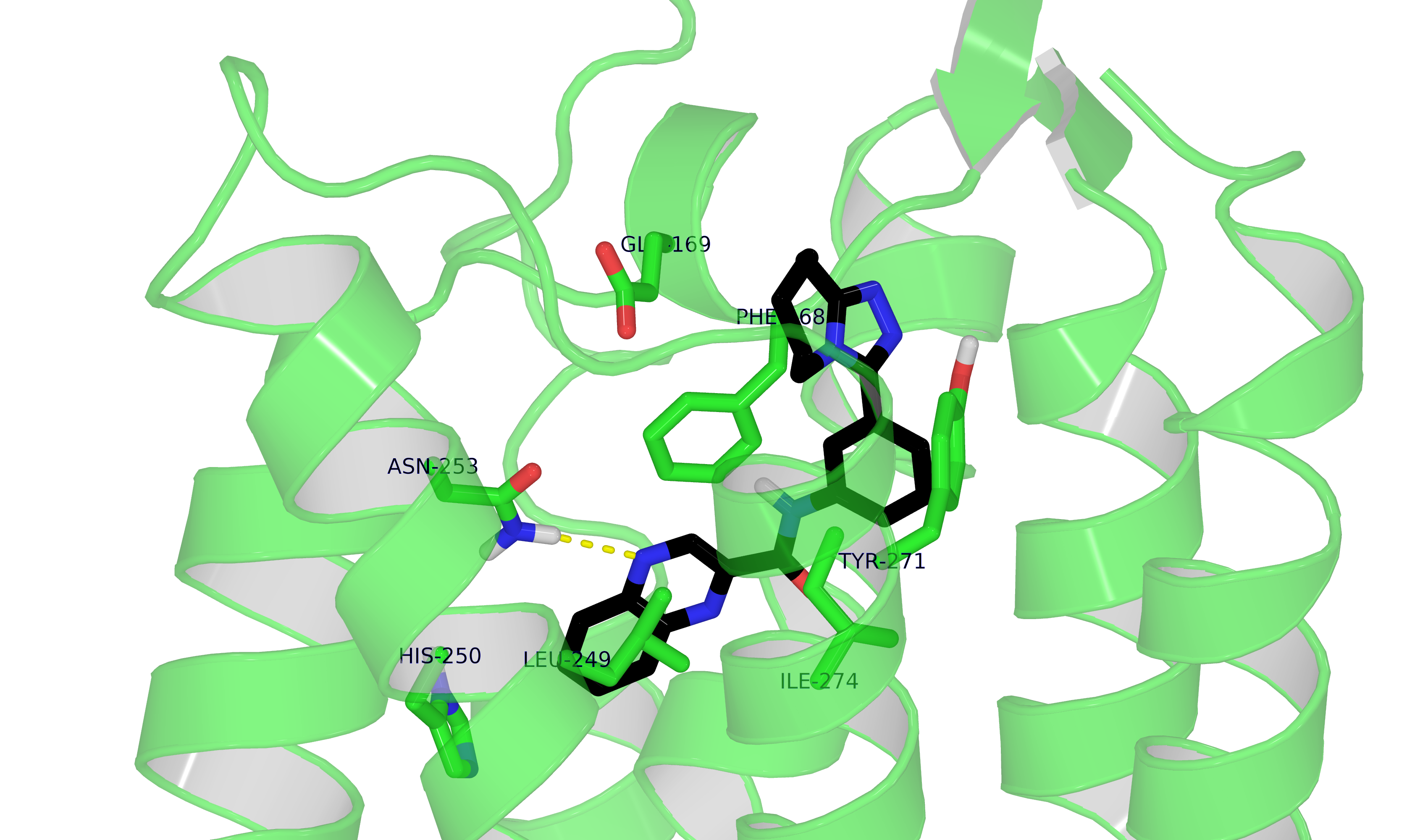

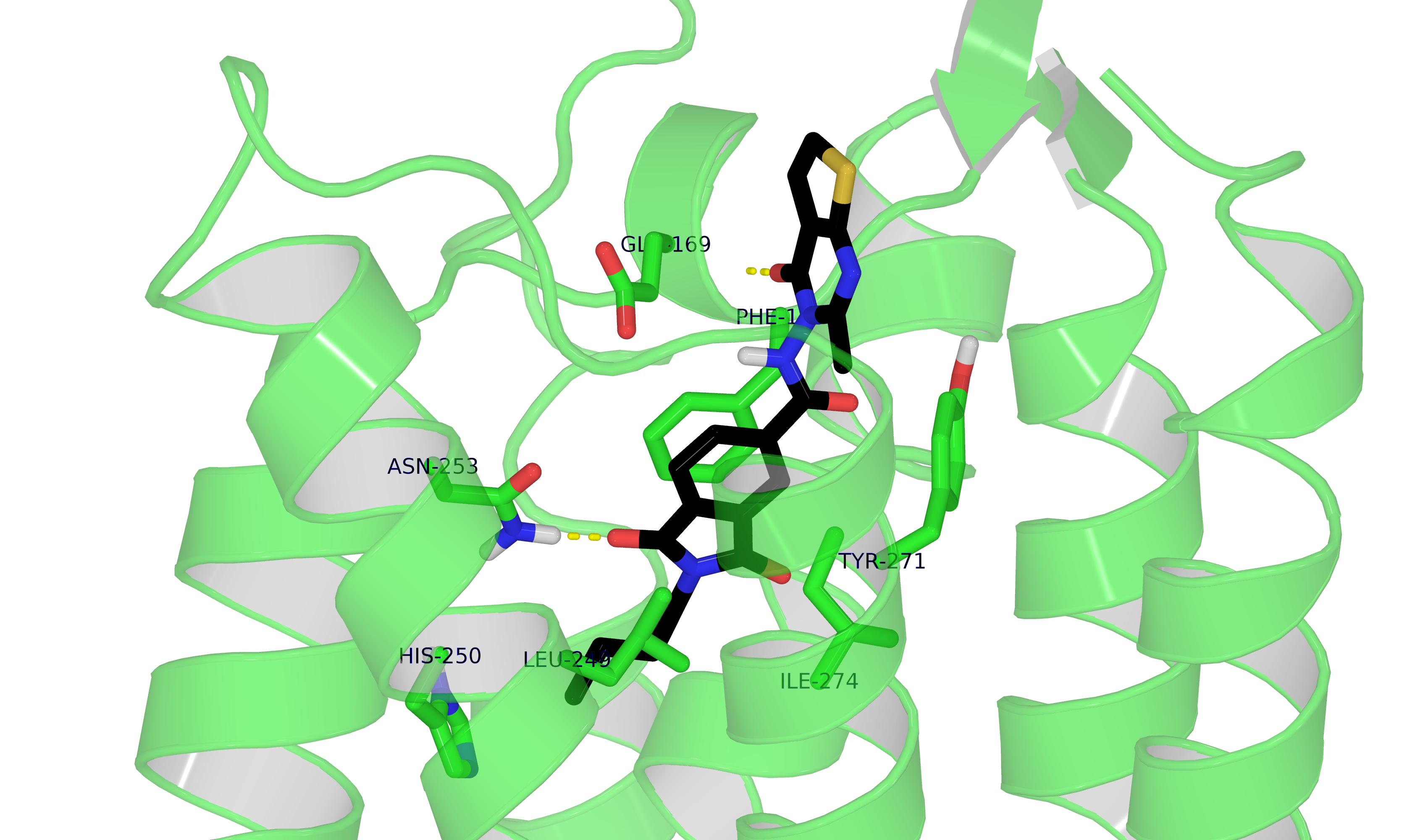


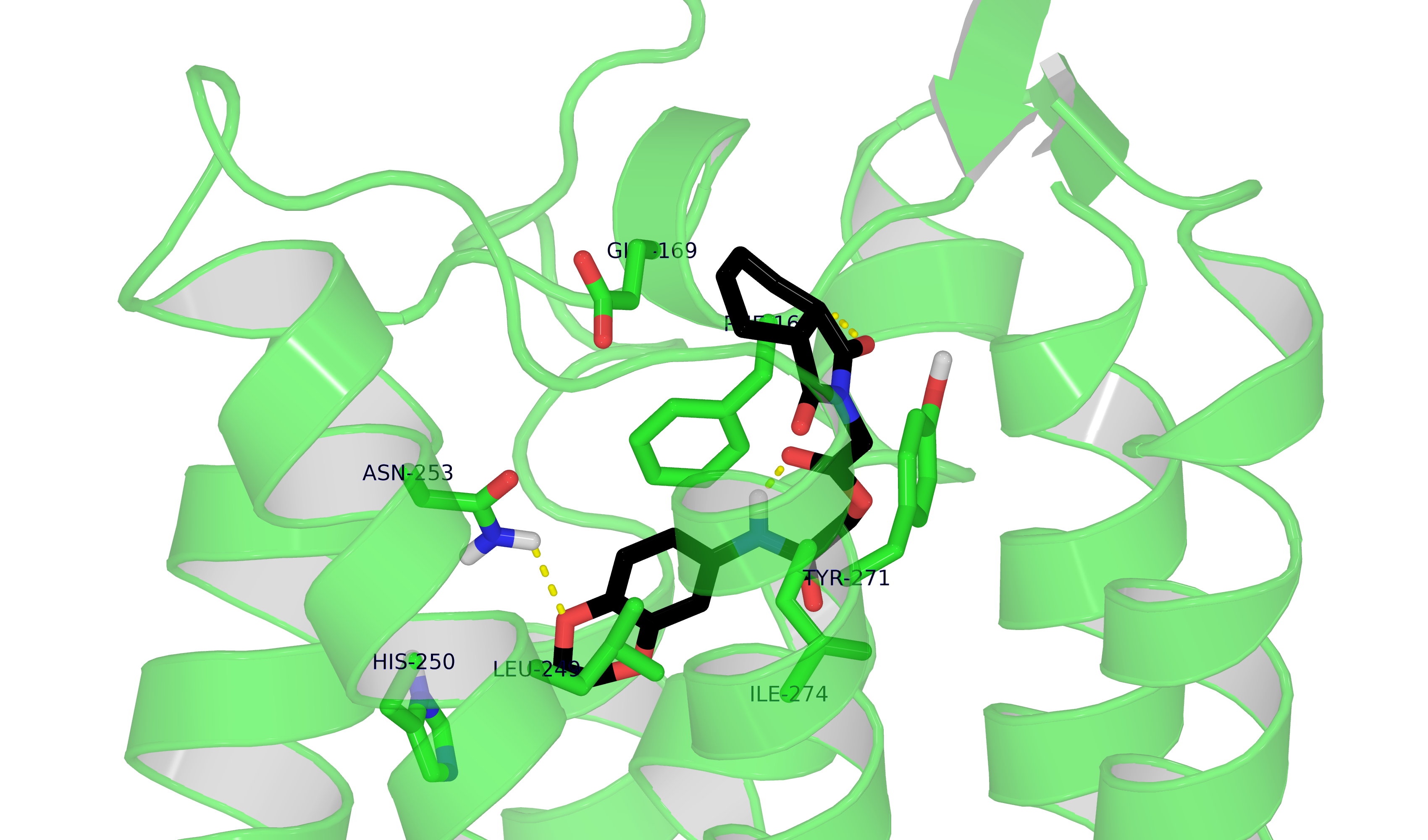


SI Figure 3, LC-MS spectrum of the first active compound.
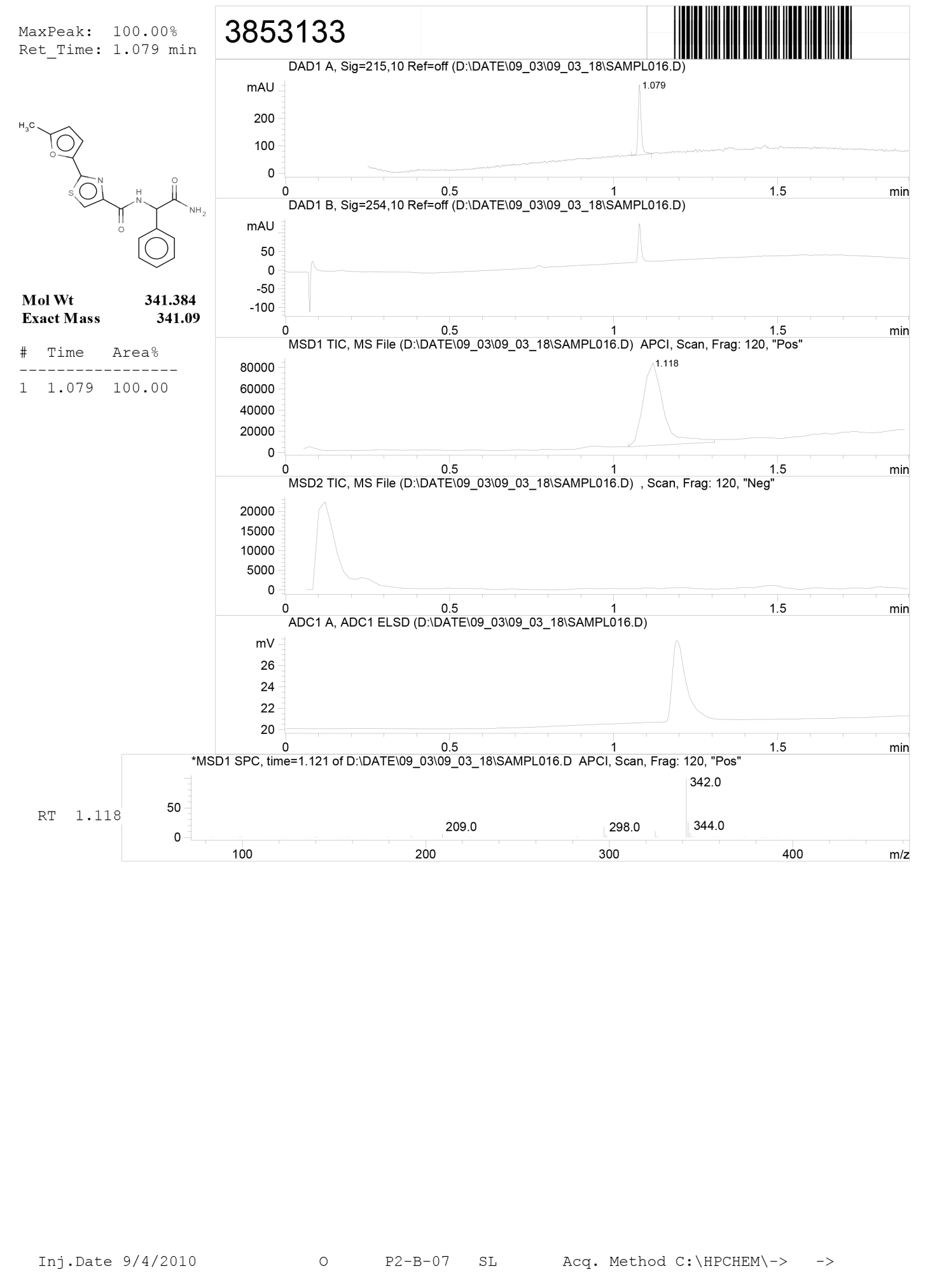


SI Figure 4, LC-MS spectrum of the second active compound.


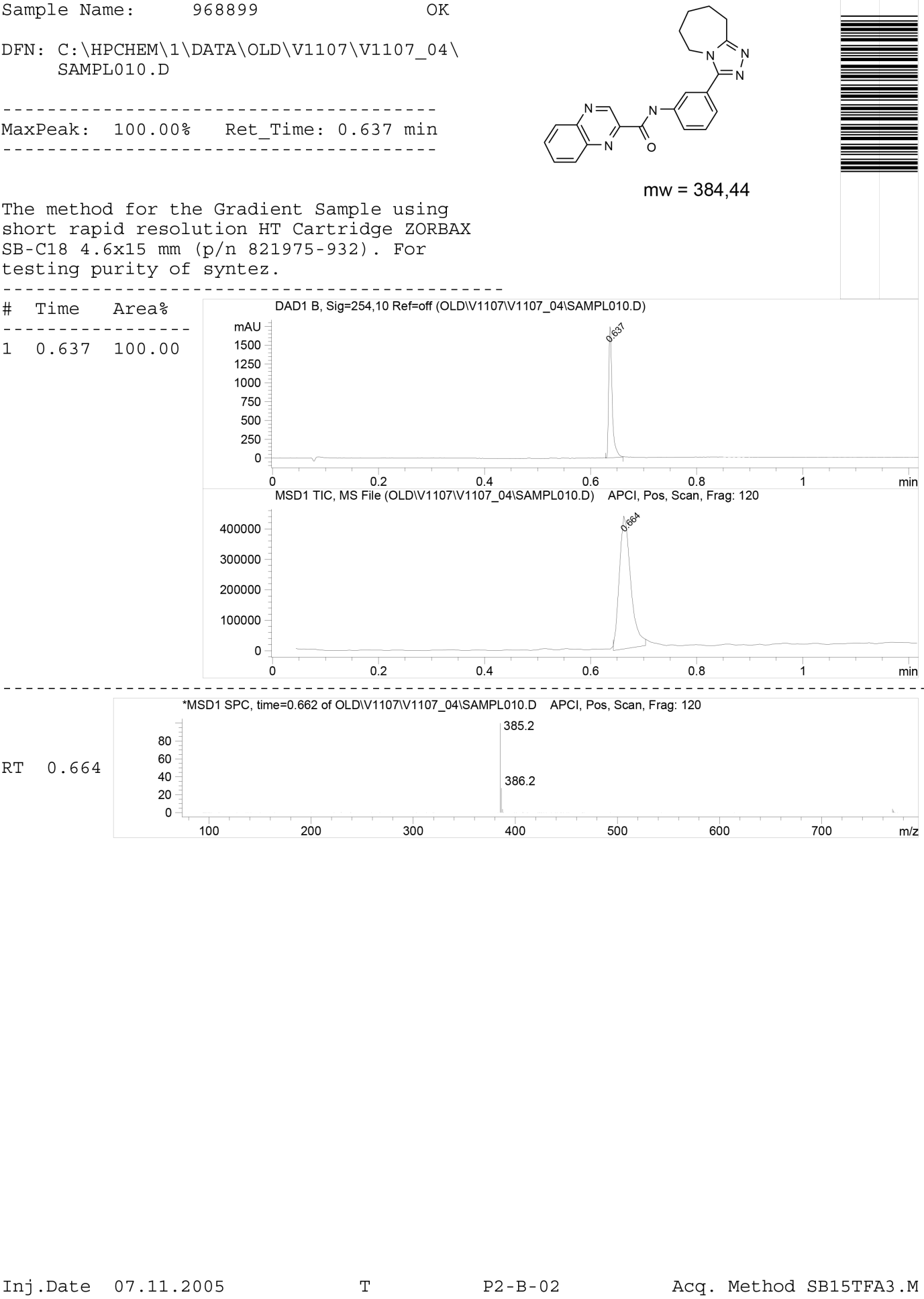


SI Figure 5, ^1^H NMR spectrum of the first active compound.


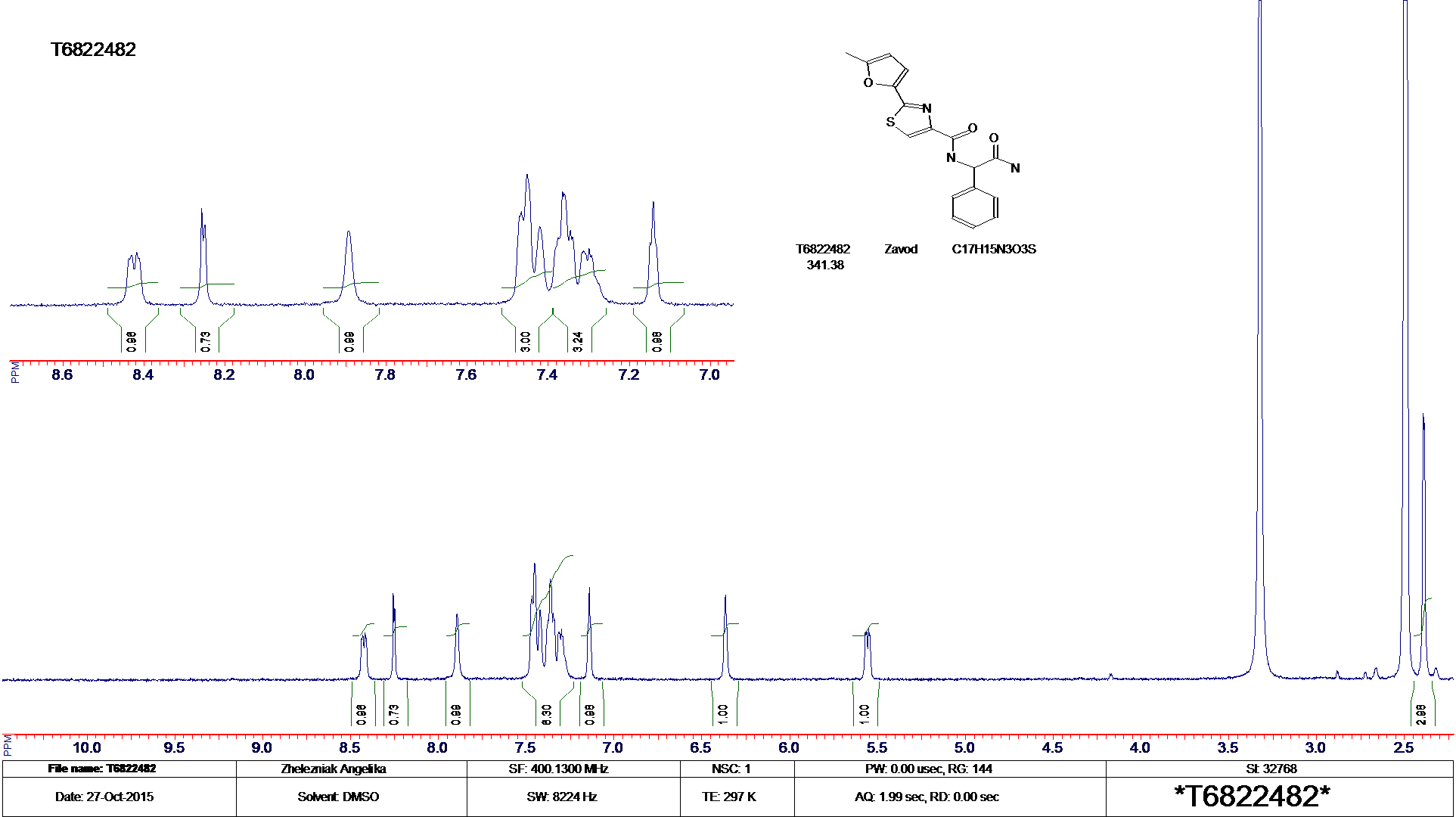


SI Figure 6, ^1^H NMR spectrum of the second active compound.


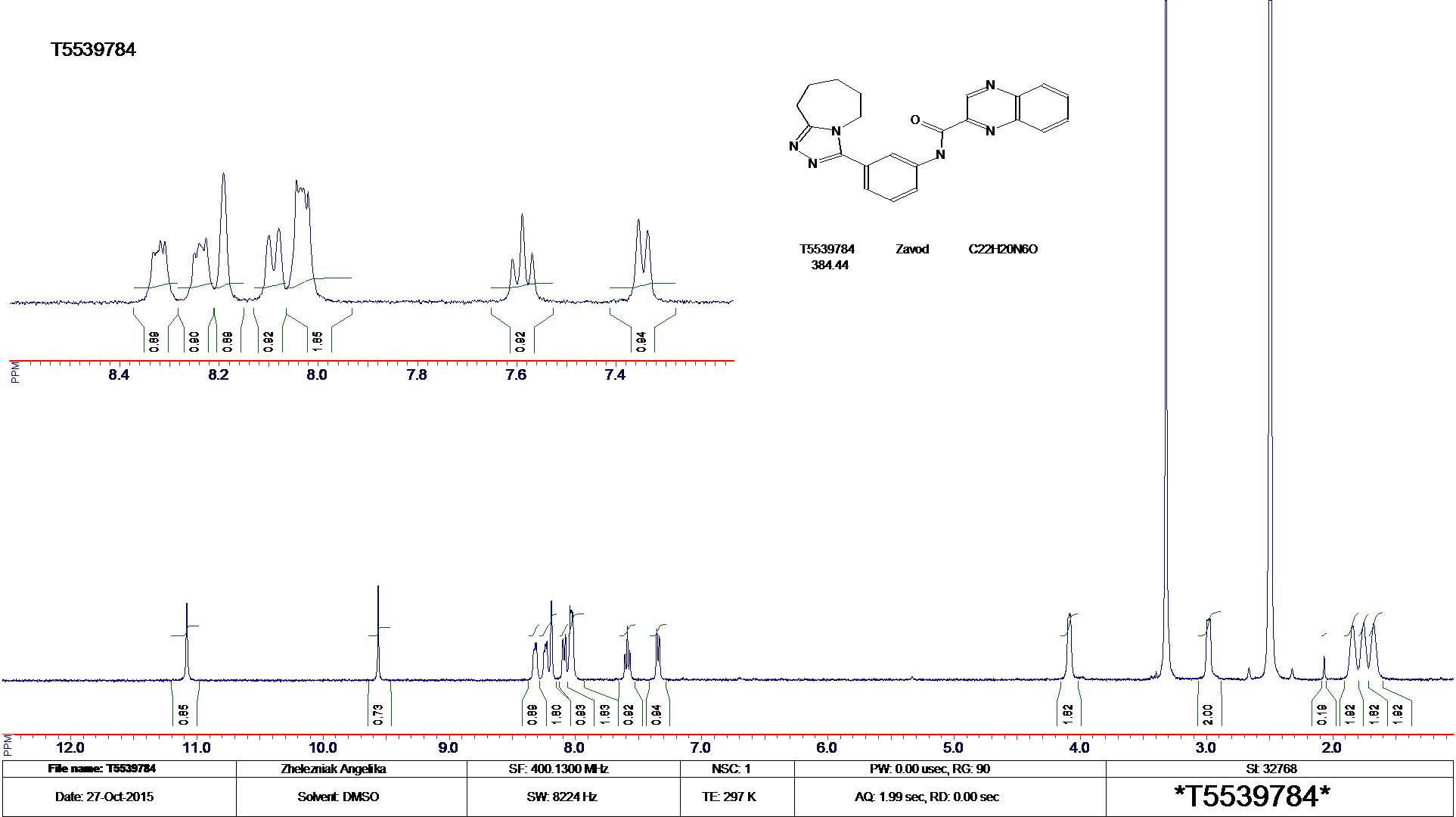

Supplement: Supplementary file 1 — Supplementary material 1 (DOCX 13675 kb) [file 10822_2016_9963_MOESM1_ESM.docx]
